# Molecular epidemiology and geographic distribution of *Theileria* spp. in Kazakhstan: a countrywide cattle survey and a southern sheep survey

**DOI:** 10.3389/fvets.2026.1987322

**Published:** 2026-10-07

**Authors:** Kassym Mukanov, Alexander Ostrovskii, Dinara Kamalova, Anara Ryskeldina, Amirkhan Kassen, Maxat Berdikulov, Sarsenbay Abdrakhmanov, Alexandr Shevtsov, Christian Bauer

**Affiliations:** 1 Laboratory of Applied Genetics, National Center of Biotechnology, Astana, Kazakhstan; 2 National Veterinary Reference Center, Astana, Kazakhstan; 3 S. Seifullin Kazakh Agrotechnical University, Astana, Kazakhstan; 4 Institute of Parasitology, Justus Liebig University Giessen, Giessen, Germany

**Keywords:** cattle, genetic diversity, Kazakhstan, molecular epidemiology, sheep, *Theileria*

## Abstract

**Background:**

Theileriosis is endemic in Central Asia; however, the geographic distribution and genetic diversity of *Theileria* spp. in livestock in Kazakhstan remain poorly characterized. This study aimed to determine the occurrence, geographic distribution, and genetic diversity of *Theileria* spp. in cattle and sheep in Kazakhstan.

**Methods:**

Blood samples from 6,224 cattle from 172 settlements across all 17 provinces of Kazakhstan and 1,467 sheep from 52 settlements in five southern provinces were collected during July–August 2022 and 2023. Samples were screened using a genus-level PCR targeting the 28S ribosomal RNA (28S rRNA) gene, followed by bidirectional sequencing for species identification. Selected *T. orientalis*-positive samples were further characterized by major piroplasm surface protein (MPSP) genotyping.

**Results:**

Among cattle, 2,142 out of 6,224 (34.4%) tested positive by PCR for at least one species of *Theileria*. *Theileria annulata*, *T. orientalis*, and *T. sinensis* were detected in 27.1, 15.1, and 0.5% of cattle, respectively. The simultaneous detection of two or three species of *Theileria* was recorded in 508 of 2,142 (23.7%) PCR-positive cattle. *Theileria orientalis* was detected in all 17 surveyed provinces, *T. annulata* in 16, and *T. sinensis* in five. MPSP analysis identified *T. orientalis* genotypes 1 (“Chitose”), 2 (“Ikeda”), and 3 (“Buffeli”). Among sheep, 169 out of 1,467 (11.5%) tested positive by PCR; *T. ovis*, *Theileria* sp. genotype OT3, and *T. annulata* were detected in 5.5, 4.4, and 2.6% of the animals examined, respectively. *T. orientalis* genotype 2 (“Ikeda”), *T. sinensis*, and *Theileria* sp. genotype OT3 were detected in Kazakhstan for the first time.

**Conclusion:**

The study demonstrated a widespread but geographically heterogeneous detection of *Theileria* species among sampled cattle across Kazakhstan and enabled the first molecular characterization of infections caused by *Theileria* species in sheep in that country. The detection of *T. orientalis* genotype 2 (“Ikeda”), *T. sinensis*, and *Theileria* sp. genotype OT3 expands the known diversity of *Theileria* species circulating in Central Asia. Since sampling was not conducted on a probabilistic basis, the observed PCR positivity should not be interpreted as representative of *Theileria* prevalence in the national livestock population. The data obtained serve as a starting point for targeted molecular surveillance of livestock and their associated tick vectors.

## 1 Introduction

Theileriosis comprises a group of tick-borne infections of domestic and wild ruminants caused by protozoa of the genus *Theileria*. The biological and veterinary significance of these parasites differs markedly among species and genotypes. *Theileria annulata*, the causative agent of tropical theileriosis, is a major pathogenic species of cattle and can cause fever, lymphadenopathy, anemia, production losses, and mortality, whereas *T. lestoquardi* is an important pathogen of sheep and goats. Other members of the genus, including *T. ovis* and many genotypes within the *T. orientalis* complex, are typically associated with persistent or subclinical infections. However, the pathogenic potential of the *T. orientalis* complex is heterogeneous, and genotype 2 (“Ikeda”) has become one of the main causes of clinical oriental theileriosis in cattle in several regions of the world (1, 2).

The epidemiology of theileriosis is closely associated with the geographic distribution and ecology of competent vectors–ticks of the family Ixodidae. Kazakhstan is of particular importance in this context because of its geographic position at the interface between Europe and Central Asia and its extensive livestock production and movement across ecologically diverse regions. Livestock movement within the country and across regional production systems may facilitate the introduction and spread of *Theileria* species and their associated tick vectors between areas with different ecological conditions. At the same time, Kazakhstan’s vast territory encompasses distinct climatic and ecological gradients–ranging from arid desert and semiarid zones in the south and west to steppe, forest-steppe, and mountainous landscapes in the northern and eastern regions (3). A wide range of ticks from the family Ixodidae has been recorded throughout the country, including species of the genera *Hyalomma*, *Dermacentor*, *Rhipicephalus*, *Haemaphysalis*, and *Ixodes*; in addition, the molecular detection of a number of tick-borne pathogens has been confirmed (4–6). The results of a recent ecological modeling study also show that the suitability of Kazakhstan’s territory for the dominant tick species varies spatially and may change under future climate change and land-use scenarios (7). These geographic, livestock-movement, and ecological factors may contribute to regional variation in *Theileria* transmission and make Kazakhstan an important setting for investigating the circulation and diversity of these parasites.

Despite the importance of livestock production in Kazakhstan, the molecular epidemiology and genetic diversity of *Theileria* spp. remain incompletely characterized. Historically, theileriosis was investigated primarily through clinical observations and microscopic examination of blood smears, and infections presumed to be caused by bovine and ovine *Theileria* spp. have been reported in Kazakhstan for several decades (8, 9). More recent molecular and serological studies have confirmed the presence of *T. annulata, T. orientalis*, and other piroplasmids in cattle and ticks, but these investigations were geographically restricted or focused on particular subsets of the livestock population (5, 10–12). Thus, although the presence of several *Theileria* taxa in Kazakhstan is established, important gaps remain regarding their countrywide occurrence, geographic heterogeneity, species composition and co-detection patterns, the diversity of *T. orientalis* genotypes, and the molecular diversity of *Theileria* in sheep.

To address these gaps, we conducted two large-scale cross-sectional molecular surveys to characterize the occurrence, geographic heterogeneity, species composition, co-detection patterns, and genetic diversity of *Theileria* spp. in livestock in Kazakhstan. The cattle survey encompassed sampling locations in all 17 provinces of the country, providing broader geographic coverage than previous molecular investigations, whereas the sheep survey covered five major sheep-producing provinces of southern Kazakhstan. Species identification was based on sequencing of the 28S rRNA locus, and selected *T. orientalis*-positive samples were further characterized using the major piroplasm surface protein (MPSP) gene. Thus, this study provides a comprehensive molecular assessment of *Theileria* spp. in sampled cattle across Kazakhstan and the first systematic molecular characterization of *Theileria* infections in sampled sheep in the country, while also documenting the geographic heterogeneity of parasite detection and the diversity of circulating taxa and *T. orientalis* genotypes. These data establish a molecular baseline for future risk-based surveillance of theileriosis in Kazakhstan.

## 2 Materials and methods

### 2.1 Study design

Two cross-sectional molecular surveys were conducted during July–August 2022 and 2023. The cattle survey included sampling locations across all 17 provinces of Kazakhstan, whereas the sheep survey covered five southern provinces. The individual animal was the unit of laboratory testing, and the settlement was considered the primary geographic sampling unit. The primary outcome was PCR positivity for at least one *Theileria* species; species-specific detection and co-detection patterns were evaluated as secondary outcomes.

### 2.2 Study area and sites

A cattle survey was conducted in 172 settlements across 94 districts in all 17 provinces of Kazakhstan (Supplementary Table S1). Sheep were sampled in 52 settlements across 31 districts in five southern provinces (Kyzylorda, Turkistan, Jambyl, Almaty, and Jetisu), which together account for approximately two-thirds of the national sheep population (Supplementary Table S2).

Livestock production in Kazakhstan is dominated by smallholder and household farming systems, which account for approximately 90% of animal husbandry practices (13). Animals are typically managed under extensive grazing conditions during the tick activity season, while routine use of acaricides or anti-*Theileria* treatments is uncommon. Kazakhstan encompasses diverse climatic zones ranging from arid deserts to continental steppe and alpine environments (3).

### 2.3 Sampling

Blood samples were collected during summer (July–August) of 2022 and 2023 as part of broader hemopathogen monitoring programs (14, 15). Settlements were selected using a non-probabilistic, convenience-based approach, depending on access to livestock holdings and voluntary participation of livestock owners. One herd was sampled within each selected settlement, and approximately 10% of eligible adult animals within each herd were sampled where feasible. Thus, each settlement contributed one herd, and herd-level and settlement-level clustering were coincident. Sampling intensity varied among provinces and was not proportional to provincial livestock population size, as the primary objective was to achieve broad geographic coverage. Therefore, the number of animals sampled in each province did not necessarily reflect local livestock density or the relative size of the provincial livestock population. All sampled animals were clinically healthy females older than 3 years. This age threshold was selected to ensure that the sampled animals had experienced at least two tick-activity/grazing seasons and, therefore, had sufficient opportunity for natural exposure to *Theileria* spp. Female animals were selected because they constitute the predominant component of the breeding livestock population in Kazakhstan and are commonly retained in herds for multiple production seasons. Accordingly, the source population comprised adult female animals from participating holdings, rather than the entire cattle or sheep population of Kazakhstan.

A total of 6,224 cattle and 1,467 sheep were included in the study. Cattle consisted mainly of crossbred animals derived from Red Steppe, Alatau, Black-and-White, and Simmental breeds, whereas sheep represented local mixed breeds. Blood samples were transported at 4 °C and processed within 48 h. Leukocyte pellets were stored at −80 °C after erythrocyte lysis.

The study protocol was approved by the Ethics Committee of the National Center for Biotechnology LLP, Kazakhstan (Protocol No. 4, October 24, 2023), and animal owners provided permission for blood sampling.

### 2.4 DNA extraction

Total DNA was extracted from 300 μL of whole blood using the previously described silica-based protocol (14), preceded by treatment with red blood cell (RBC) lysis buffer to remove erythrocytes and increase the relative concentration of nucleated cells. The resulting samples were subjected to proteinase K/SDS digestion, silica-based DNA binding, washing, and elution in TE buffer.

### 2.5 PCR and genotyping

All samples were screened for *Theileria* spp. DNA using conventional PCR targeting the 28S rRNA gene. The 28S rRNA gene was selected as a genus-level screening target because it contains conserved and variable regions that support broad amplification and sequence-based discrimination of *Theileria* spp. Previous studies have demonstrated that the 28S rRNA, particularly the D2–D3 region, is useful for phylogenetic analysis and differentiation of ruminant *Theileria* spp. (16). A novel primer pair was designed to amplify an 865–892 bp fragment: Thel_28SF (5′-GGCTAACCCATGTCCAAACATA-3′) and Thel_28SR (5′-CACGAGCCAAGGAGTCTAACACT-3′). Primer specificity was evaluated in silico using NCBI Primer-BLAST against available *Theileria*, *Babesia*, and other apicomplexan sequences in GenBank.

PCR reactions were performed in a final volume of 25 μL containing 12.5 μL BioMaster HS-Taq PCR-Special (Biolabmix, Russia), 400 nM of each primer, 5 μL DNA template, and nuclease-free water. Amplification conditions consisted of initial denaturation at 95 °C for 5 min, followed by 40 cycles of 95 °C for 20 s, 63 °C for 30 s, and 72 °C for 80 s, followed by a final extension at 72 °C for 5 min. Negative controls (nuclease-free water) and positive controls (*Theileria*-positive DNA) were included in all runs. PCR products were visualized by electrophoresis on 1.5% agarose gels stained with ethidium bromide using a GelDoc system (Bio-Rad, USA).

Among 942 *T. orientalis*-positive cattle, 364 samples showed homogeneous 28S rRNA chromatograms and were considered eligible for MPSP genotyping. Of these, 149 samples were selected for PCR targeting a 776-bp fragment of the major piroplasm surface protein (MPSP) gene as previously described (17). Eighty-four samples yielded the expected MPSP amplicon, 57 yielded sequence data, and 31 produced homogeneous MPSP chromatograms without mixed-base positions. Seven representative sequences were selected for phylogenetic analysis and GenBank deposition. For further characterization of the ALM-7420 ovine sample, an additional nested PCR targeting the 18S rRNA gene was performed using the primer sets Thei F1/Thei R1 and Thei F2/Thei R2, as described previously (18). The outer primers were Thei F1 (5′-AACCTGGTTGATCCTGCCAG-3′) and Thei R1 (5′-AAACCTTGTTACGACTTCTC-3′), while the inner primers were Thei F2 (5′-TGATGT TCGTTTYTACATGG-3′) and Thei R2 (5′-CTAGGCATTCCTCGTTCACG-3′).

### 2.6 Sequencing and phylogenetic analysis

All amplification products from *Theileria*-positive samples were subjected to sequencing to identify the respective species. PCR products were sequenced using the BigDye Terminator v3.1 Cycle Sequencing Kit (Thermo Fisher Scientific, Vilnius, Lithuania) on an ABI 3730xl Genetic Analyzer (Applied Biosystems, USA). Sequences were assembled using SeqMan 6.1 (19). Sequence alignments were generated using ClustalW implemented in MEGA 12 (20). The Tamura–Nei nucleotide substitution model was selected according to the Bayesian information criterion. Maximum Likelihood phylogenetic trees were reconstructed using the selected model with 1,000 bootstrap replicates. Positions containing gaps and missing data were handled using complete deletion.

Mixed infections were inferred from chromatograms showing reproducible mixed-base positions at species-discriminating sites. Identification of mixed *Theileria* infections was based on comparative analysis of bidirectional Sanger sequencing chromatograms of the 28S rRNA gene. Mixed-base positions were identified using Sequencing Analysis Software v5.1 (Applied Biosystems) with a mixed-base calling threshold of 25% and were confirmed in both sequencing directions. Reference sequences for each *Theileria* species were obtained from GenBank and from confirmed single-species isolates identified in this study that showed no mixed-base positions. Mixed infections were inferred when reproducible overlapping peaks were observed at multiple diagnostic nucleotide positions that consistently differentiated two species in the reference sequence.

Only high-quality chromatogram regions were analyzed, and ambiguous bases within low-quality terminal regions were excluded. Because the analyzed 28S rRNA fragment contained species-specific polymorphisms, concordant mixed-base signals at multiple diagnostic positions were interpreted as evidence of coinfection rather than sequencing artifacts. Low-abundance co-infections may have remained undetected because Sanger sequencing has limited sensitivity for minor sequence variants. Only clearly discernible mixed signals were considered indicative of mixed infection.

### 2.7 Statistical analysis

PCR positivity was calculated as the proportion of PCR-positive animals among all animals tested and is reported with exact 95% confidence intervals calculated using the Clopper–Pearson method. Analyses were conducted separately for cattle and sheep and for each *Theileria* taxon. Overall differences in PCR positivity among provinces were evaluated using Pearson’s chi-square test. Because of sparse cell counts for *T. sinensis* in cattle, provincial differences for this species were evaluated using Fisher’s exact test for an R × C contingency table with Monte Carlo estimation of the *p*-value (1,000,000 replicates). The association between *T. annulata* and *T. orientalis* detection in cattle was evaluated using an odds ratio (OR) with a 95% confidence interval based on a 2 × 2 contingency table; statistical significance was assessed using Fisher’s exact test.

Geographic associations in cattle were evaluated using binomial generalized estimating equations (GEE) with a logit link. Latitude was selected as a continuous geographic predictor because it represents a broad north–south climatic and ecological gradient across Kazakhstan, along which the composition of ixodid tick populations varies. Previous studies have documented substantial geographic variation in tick distributions and tick-borne pathogens in Kazakhstan, including *Theileria* spp. (4–6), while recent habitat-suitability modelling indicates that climate and land-use changes may alter the distribution of several dominant tick species in the country (7). Longitude was not included as a primary geographic predictor, because the prespecified analysis focused on the north–south ecological gradient. Environmental and tick-related covariates were not incorporated because comparable data on tick abundance, grazing conditions, and herd management were not available for all sampling locations. Settlement was specified as the clustering unit using an exchangeable working correlation structure to account for within-settlement correlation among animals; because one herd was sampled per settlement, this also corresponded to the herd-level clustering structure. Separate models were fitted for overall *Theileria* spp. detection and for *T. annulata*, *T. orientalis*, and *T. sinensis*. Model estimates are reported as ORs per 1° northward increase in latitude with 95% confidence intervals. All statistical tests were two-sided, with *p* < 0.05 considered statistically significant. Analyses were performed using Python 3.13.5 with statsmodels version 0.14.6 and SciPy version 1.17.0.

### 2.8 Data availability

All sequences generated in this study have been deposited in GenBank under accession numbers PZ312048–PZ312062, PZ312064–PZ312066, and PZ319530–PZ319536. Detailed information on sample identifiers, target genes, and corresponding accession numbers is provided in Supplementary Table S3.

## 3 Results

### 3.1 *Theileria* spp. in cattle

Overall, 2,142/6,224 cattle (34.4%; 95% CI, 33.2–35.6) were PCR-positive for at least one *Theileria* species. *Theileria annulata* was detected in 1,685/6,224 (27.1%), *T. orientalis* in 942/6,224 (15.1%), and *T. sinensis* in 33/6,224 (0.5%) cattle (Table 1). Among PCR-positive cattle, 1,634/2,142 (76.3%) showed single-species detection, whereas 508/2,142 (23.7%) showed co-detection of two or three *Theileria* species. Concurrent detection of *T. annulata* and *T. orientalis*, including triple-species detections, occurred in 491/6,224 (7.9%) cattle. The odds of *T. orientalis* detection were higher among *T. annulata*-positive than among *T. annulata*-negative cattle (OR, 3.73; 95% CI, 3.23–4.30; *p* < 0.0001), indicating an epidemiological association between detection of the two taxa.

**Table 1:** PCR positivity for *Theileria* spp. in 6,224 cattle across all 17 provinces of Kazakhstan, determined by PCR screening followed by sequencing for species identification.

*Theileria* sp.	N positive	% positive (CI)^a^
Any *Theileria* spp.	2,142	34.4 (33.2–35.6)
*T. annulata*	1,685	27.1 (26.0–28.2)
*T. orientalis*	942	15.1 (14.3–16.0)
*T. sinensis*	33	0.5 (0.4–0.7)
Mono-infection	1,634	76.3^b^
*T. annulata*	1,192	70.7^c^
*T. orientalis*	436	46.3^c^
*T. sinensis*	6	18.2^c^
Co-infection	508	23.7^b^
*T. annulata* + *T. orientalis*	481	22.5^b^
*T. annulata* + *T. sinensis*	2	0.09^b^
*T. orientalis* + *T. sinensis*	15	0.7^b^
*T. annulata* + *T. orientalis* + *T. sinensis*	10	0.5^b^

^a^95% confidence intervals.

^b^Percentages are based on all PCR-tested positive cattle.

^c^Percentages are based on cattle that tested positive for the respective *Theileria* species.

PCR-positive cattle were identified in 123/172 (71.5%) surveyed settlements, whereas no PCR-positive animals were detected in 49/172 (28.5%) settlements. Overall PCR positivity differed significantly among provinces (χ^2^ = 1697.27, df = 16, *p* < 0.0001), ranging from 0.4% in Karaganda province to 66.3% in Turkistan province. High PCR positivity was also recorded in Jambyl (61.9%), Kyzylorda (58.8%), and Almaty (53.4%) provinces. Numbers of cattle tested varied among provinces, and the corresponding sample sizes and 95% confidence intervals for PCR positivity are provided in Supplementary Table S1. In the GEE model accounting for clustering within settlements, increasing latitude was inversely associated with overall *Theileria* spp. detection, with each 1° northward increase associated with lower odds of PCR positivity (OR, 0.78; 95% CI, 0.71–0.85; *p* < 0.001) (Supplementary Table S4).

Latitude was also inversely associated with *T. annulata* detection (OR per 1° northward increase, 0.70; 95% CI, 0.65–0.77; *p* < 0.001) and, more weakly, with *T. orientalis* detection (OR, 0.90; 95% CI, 0.82–0.99; *p* = 0.038). In contrast, *T. sinensis* detection was positively associated with latitude (OR, 1.52; 95% CI, 1.10–2.10; *p* = 0.012).

*Theileria annulata* was detected in 16 provinces and predominated in the southern regions, particularly in Turkistan (66.3%), Kyzylorda (58.8%), and Jambyl (46.5%) (Figure 1A). Intermediate PCR positivity was observed in Mangystau (30.0%), Ulytau (22.0%), Almaty (17.1%), Atyrau (13.9%), and Kostanay (10.2%), whereas PCR positivity remained below 10% in most northern, northwestern, and eastern provinces. The parasite was not detected in cattle in Jetisu province.

**Figure 1:**
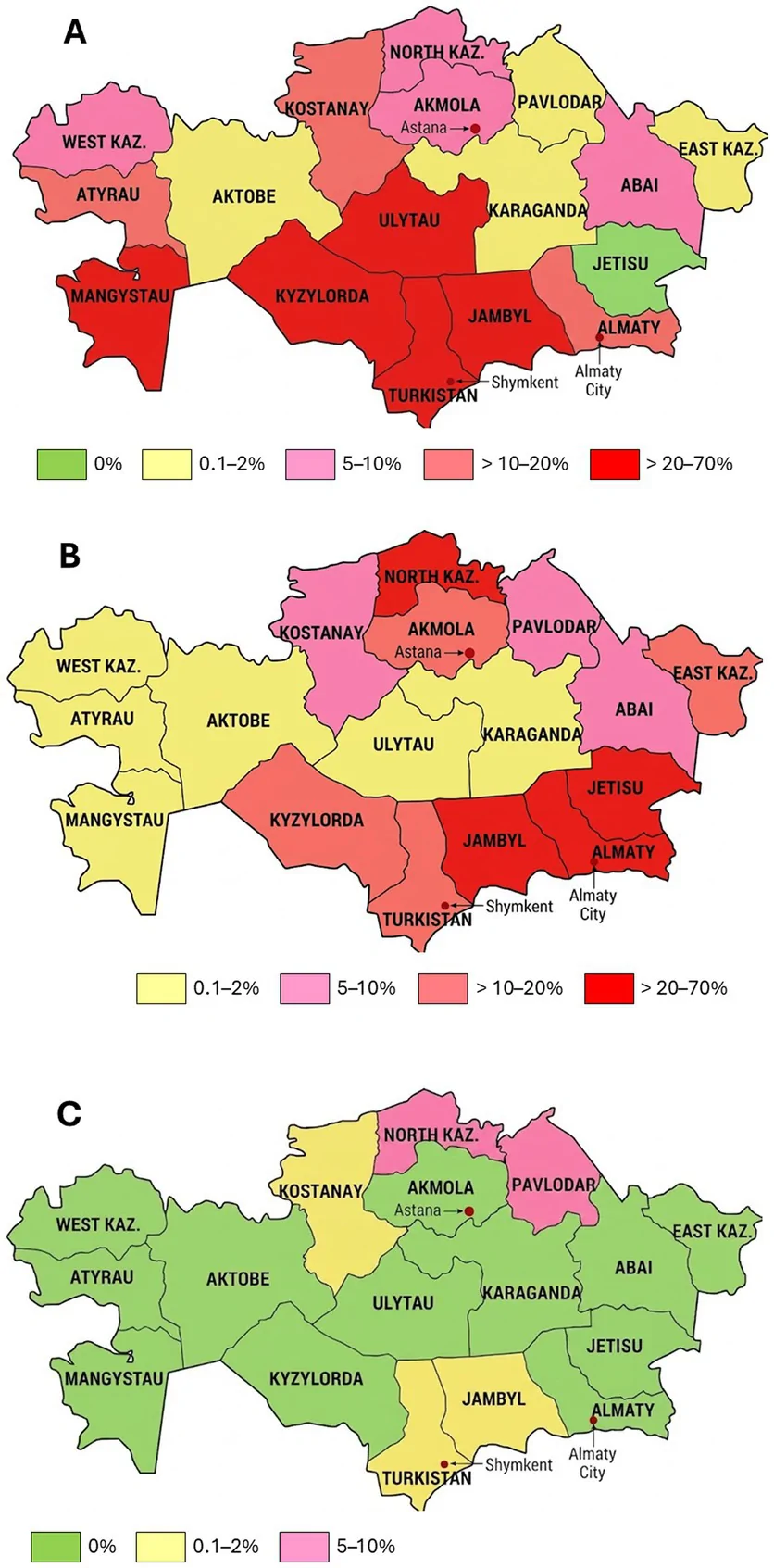
Map of Kazakhstan illustrating the provincial PCR positivity of *Theileria* spp. infections in cattle, categorized into five PCR positivity groups: **(A)** *T. annulata*, **(B)** *T. orientalis*, and **(C)** *T. sinensis*.

*Theileria orientalis* was detected across all 17 sampled provinces (Figure 1B). The highest PCR positivity was observed in Almaty (44.0%), Jetisu (36.0%), North Kazakhstan (30.9%), and Jambyl (30.3%) provinces. Values of 7.9–17.8% were recorded in Abai, Pavlodar, Kostanay, Akmola, East Kazakhstan, Kyzylorda, and Turkistan provinces, whereas positivity ranged from 0.2 to 1.1% in several western and central provinces.

*Theileria sinensis* was detected in five provinces (Figure 1C), most frequently in Pavlodar (7.3%) and North Kazakhstan (2.5%) provinces, while only sporadic detections (0.1–0.6%) were recorded in Kostanay, Turkistan, and Jambyl provinces.

Analysis of the 28S rRNA sequences identified 14 distinct sequence variants, including six assigned to *T. annulata*, seven to *T. orientalis*, and one to *T. sinensis* (Figure 2).

**Figure 2:**
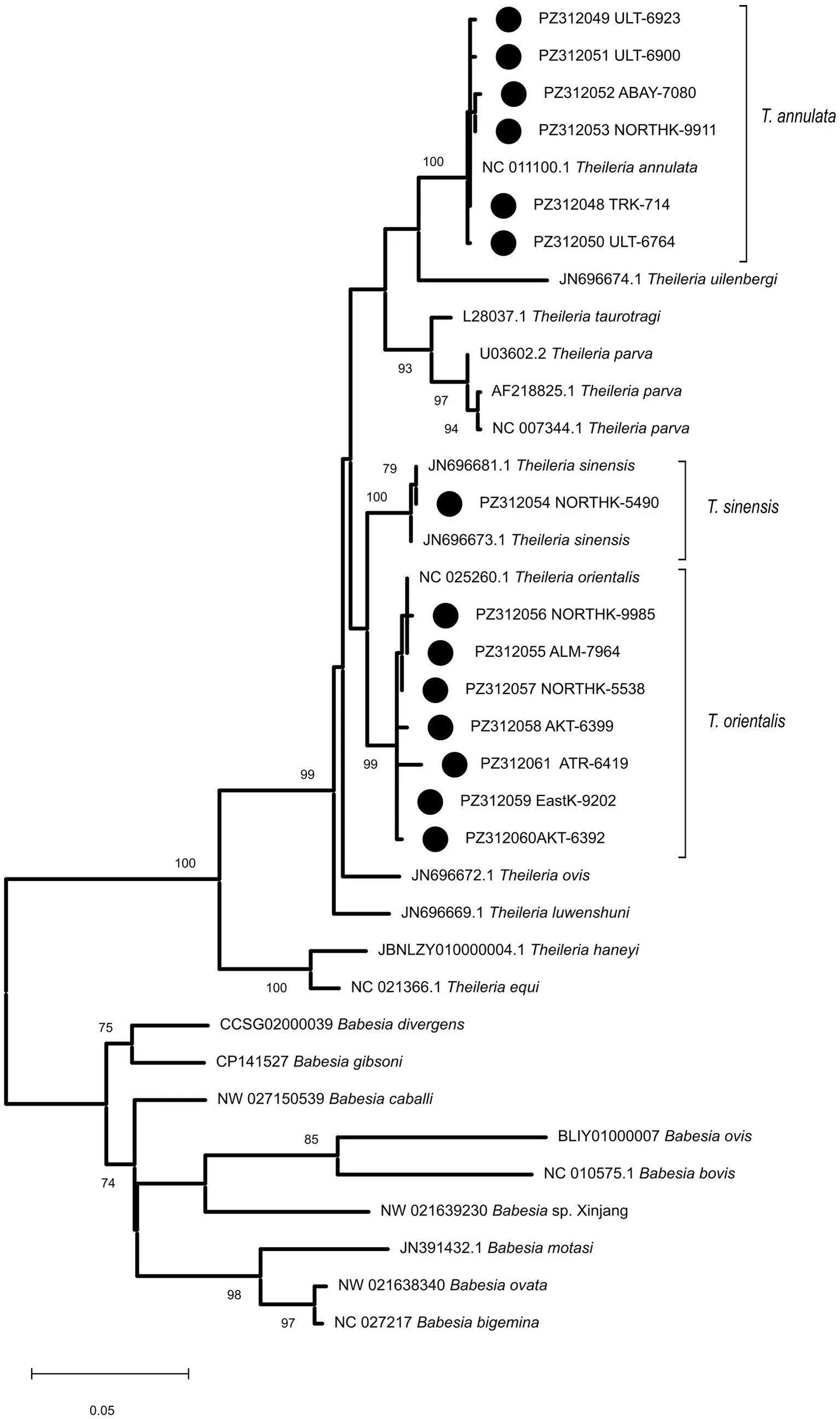
Phylogenetic tree of *Theileria* spp. based on analysis of a 665 bp fragment of the 28S rRNA sequence variants obtained from cattle in Kazakhstan, constructed using the Maximum Likelihood method in MEGA 12 software. Numbers at the nodes indicate bootstrap support values (%). The scale bar represents the number of nucleotide substitutions per site. Sequences generated in the present study are indicated by the symbol ●.

*Theileria orientalis* MPSP sequence data were obtained from 57 samples, of which 31 showed homogeneous chromatograms suitable for unambiguous sequence interpretation. Seven representative sequences were selected for phylogenetic analysis and GenBank deposition. These represented genotype 3 (“Buffeli”; four sequences), genotype 1 (“Chitose”; two sequences), and genotype 2 (“Ikeda”; one sequence) (Figure 3). Genotype 2 was represented by sample ALM-7607 from Almaty province. Thus, these results represent the *T. orientalis* genotypes detected among the selected samples and do not reflect their frequency or distribution in the cattle population of Kazakhstan.

**Figure 3:**
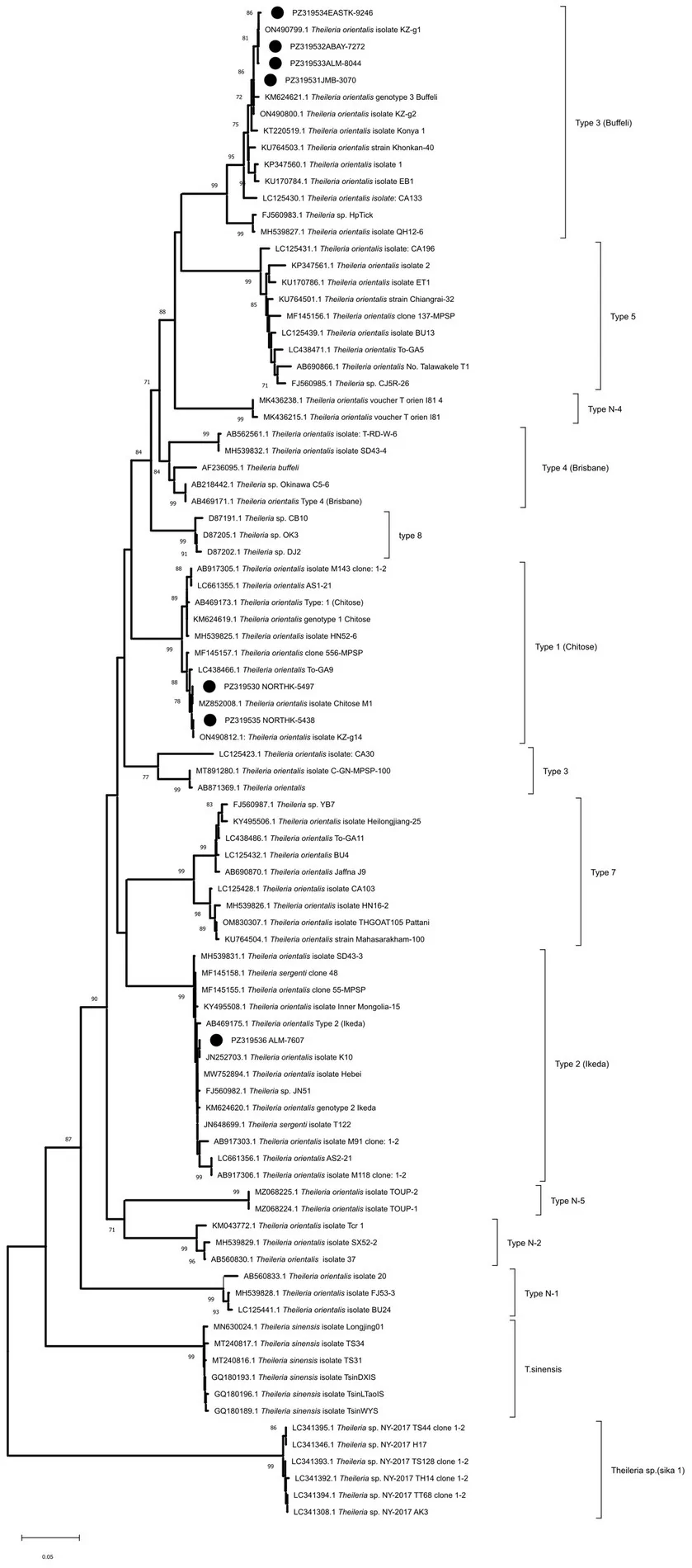
Phylogenetic tree of *T. orientalis* based on a 567-bp fragment of the MPSP gene, including seven representative sequences obtained from cattle in Kazakhstan, constructed using the Maximum Likelihood method in MEGA 12 software. Numbers at the nodes indicate bootstrap support values (%). The scale bar represents the number of nucleotide substitutions per site. Sequences generated in the present study are indicated by the symbol ●.

### 3.2 *Theileria* spp. in sheep

Overall, 169/1,467 sheep (11.5%; 95% CI, 9.9–13.3) were PCR-positive for at least one *Theileria* species. *Theileria ovis* was detected in 80/1,467 (5.5%; 95% CI, 4.3–6.7), *Theileria* sp. genotype OT3 in 65/1,467 (4.4%; 95% CI, 3.4–5.6), and *T. annulata* in 38/1,467 (2.6%; 95% CI, 1.8–3.5) sheep (Table 2). Among PCR-positive sheep, 155/169 (91.7%) showed single-species detection, whereas 14/169 (8.3%) showed co-detection of two *Theileria* taxa. Co-detection of *T. ovis* and *T. annulata* occurred in 12/169 (7.1%) positive sheep, and co-detection of *T. annulata* and *Theileria* sp. genotype OT3 occurred in 2/169 (1.2%).

**Table 2:** PCR positivity for *Theileria* spp. in 1,467 sheep from five southern provinces of Kazakhstan, determined by PCR screening followed by sequencing for species identification.

*Theileria* sp.	N positive	% positive (CI)^a^
Any *Theileria* spp.	169	11.5 (9.9–13.3)
*T. ovis*	80	5.5 (4.3–6.7)
*T. annulata*	38	2.6 (1.8–3.5)
*Theileria* genotype OT3	65	4.4 (3.4–5.6)
Mono-infection	155	91.7^b^
*T. ovis*	68	85.0^c^
*T. annulata*	24	63.2^c^
*Theileria* sp. genotype OT3	63	96.9^c^
Co-infection	14	8.3^b^
*T. ovis* + *T. annulata*	12	7.1^b^
*T. annulata* + *Theileria* sp. genotype OT3	2	1.2^b^

^a^95% confidence intervals.

^b^Percentages are based on all PCR-tested positive sheep.

^c^Percentages are based on sheep that tested positive for the respective *Theileria* species.

PCR-positive sheep were identified in 21/52 (40.4%) surveyed settlements. Overall PCR positivity differed significantly among provinces (χ^2^ = 150.45, df = 4, *p* < 0.0001), with the highest values recorded in Kyzylorda (22.6%) and Almaty (22.3%) provinces, followed by Turkistan (5.1%), Jambyl (1.1%), and Jetisu (1.0%) provinces (Supplementary Table S2).

*Theileria ovis* was detected in all five surveyed provinces, with provincial PCR positivity ranging from 0.3% in Jetisu to 16.6% in Kyzylorda province. *Theileria annulata* was also detected in all five provinces, ranging from 0.3% in Almaty to 7.3% in Kyzylorda province. In contrast, *Theileria* sp. genotype OT3 was detected only in Almaty (21.6%) and Jetisu (0.7%) provinces.

Phylogenetic analysis of the 28S rRNA sequences obtained from sheep confirmed the assignment of the respective sequences to *T. ovis* and *T. annulata* (Figure 4). The 28S rRNA sequence obtained from sample ALM-7420 clustered close to *T. ovis* sequences; however, reference 28S rRNA sequences assigned to *Theileria* sp. genotype OT3 were unavailable for direct comparison. Analysis of an additional 1,174-bp 18S rRNA sequence from ALM-7420 placed the sequence within the *Theileria* sp. OT3 clade (Figure 5). The ALM-7420 18S rRNA sequence showed 100% identity to OT3 sequences included in the comparative analysis (Figure 6).

**Figure 4:**
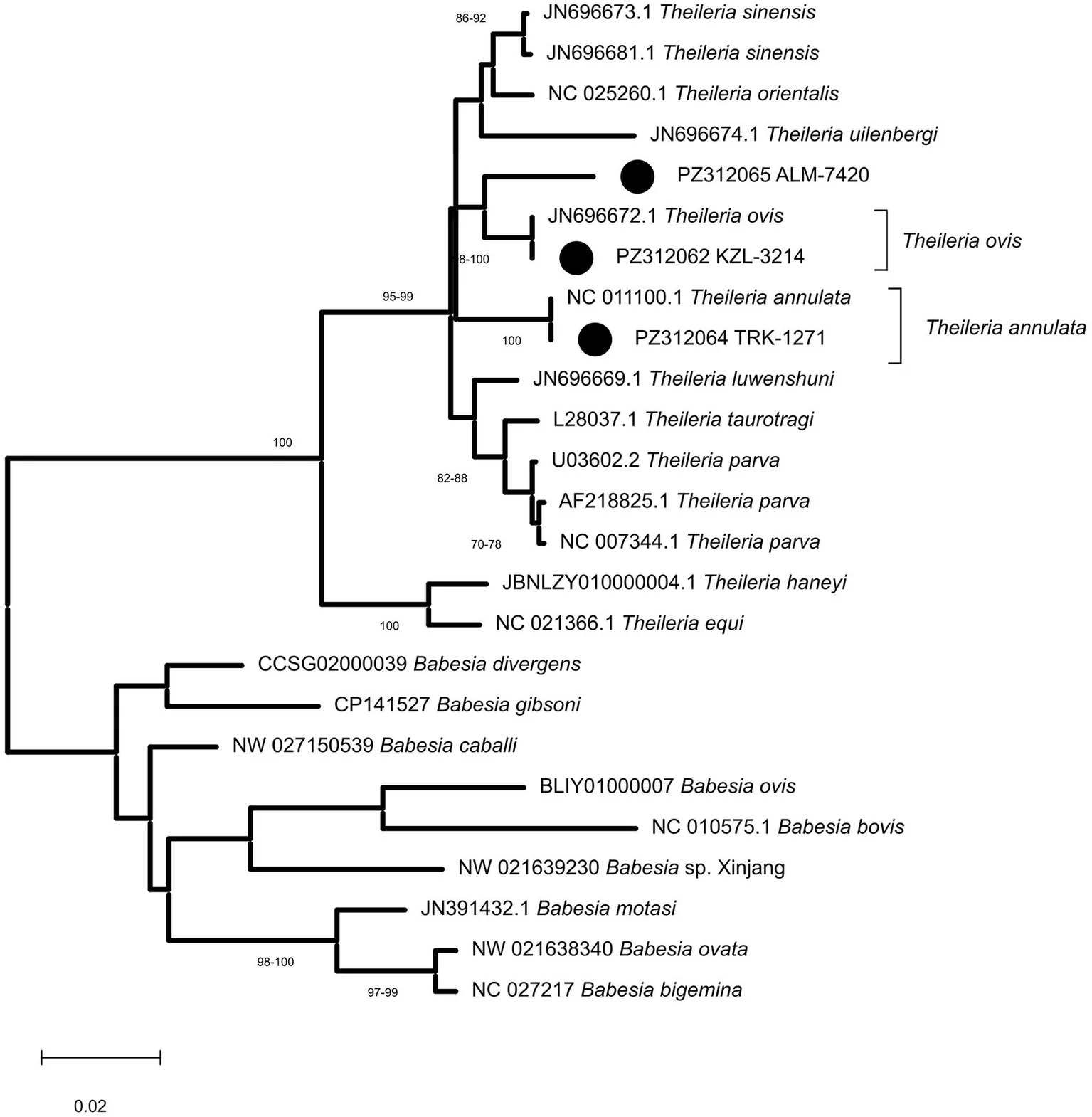
Phylogenetic tree of *Theileria* spp. based on analysis of a 665 bp fragment of the 28S rRNA gene from three sheep genotypes, constructed using the Maximum Likelihood method in MEGA 12 software. Numbers at the nodes indicate bootstrap support values (%). The scale bar represents the number of nucleotide substitutions per site. Sequences generated in the present study are indicated by the symbol ●.

**Figure 5:**
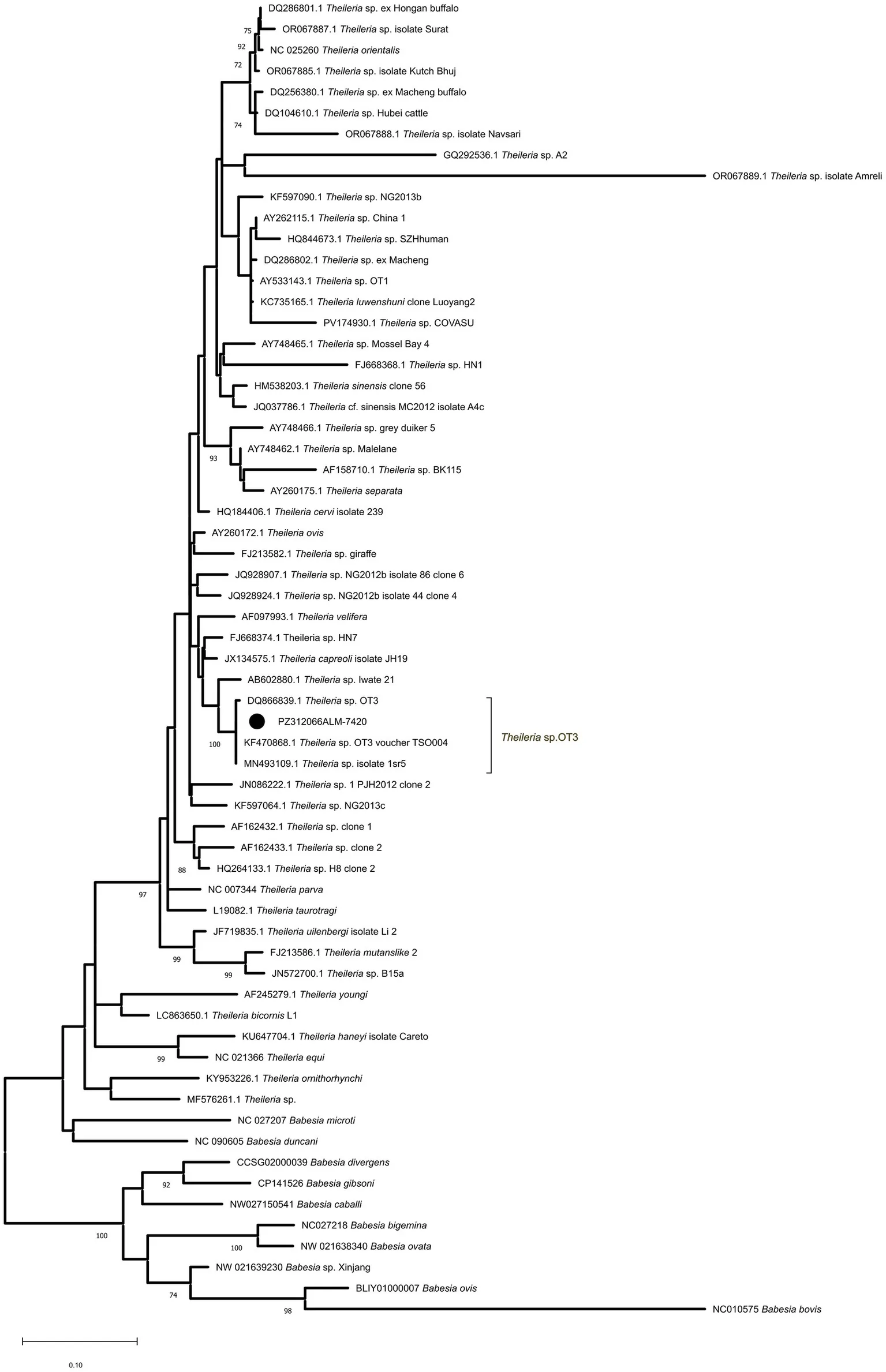
Phylogenetic tree of *Theileria* spp. based on analysis of a 1,174 bp fragment of the 18S rRNA gene from a genotype obtained from sheep in Almaty province, constructed using the Maximum Likelihood method in MEGA 12 software. Numbers at the nodes indicate bootstrap support values (%). The scale bar represents the number of nucleotide substitutions per site. The sequence generated in the present study is indicated by the symbol ●.

**Figure 6:**
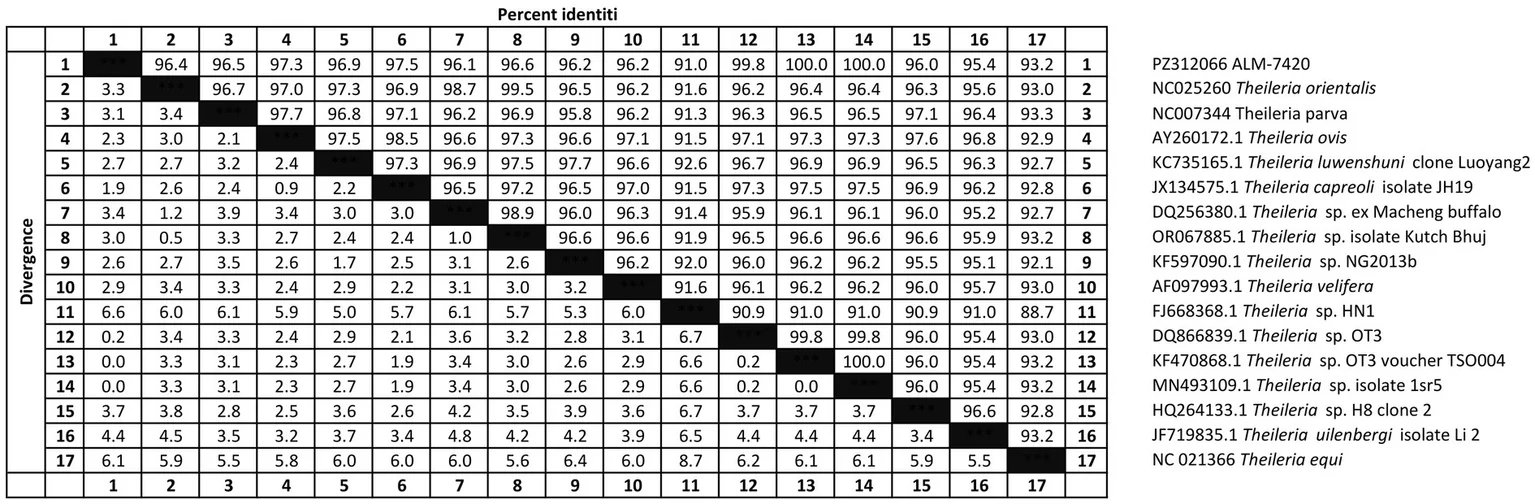
Sequence identity and distance matrix based on 18S rRNA gene sequences of *Theileria* sp. genotype OT3 detected in sheep from Almaty province compared with closely related *Theileria* species and outgroup clades.

## 4 Discussion

The present study provides the first geographically extensive molecular survey of *Theileria* spp. in cattle, encompassing all 17 provinces of Kazakhstan, as well as the first molecular characterization of *Theileria* infections in sheep in the country. Previous molecular investigations in Kazakhstan were geographically limited and focused predominantly on bovine infections (10, 21). In cattle*, T. annulata* and *T. orientalis* were widely detected, whereas *T. sinensis* occurred at substantially lower frequency and was restricted to five provinces. In sheep, *T. ovis*, *T. annulata*, and *Theileria* sp. genotype OT3 were identified, with marked differences in their geographic distributions. The study also provides the first molecular evidence of *T. orientalis* genotype 2 (“Ikeda”), *T. sinensis*, and *Theileria* sp. genotype OT3 in Kazakhstan. The frequent co-detection of *T. annulata* and *T. orientalis* in cattle represents an epidemiological association between detection of the two taxa rather than evidence of a biological interaction. The higher odds of *T. orientalis* detection among *T. annulata*-positive cattle may reflect shared ecological or herd-level exposure to infected ticks rather than interaction between the parasites themselves. Because tick abundance, vector infection status, and relevant management factors were not assessed, the mechanisms underlying this association cannot be determined from the present data.

A prominent epidemiological feature of the present survey was the marked geographic heterogeneity in *Theileria* detection. Although PCR-positive cattle were identified in 123 of 172 surveyed settlements, positivity varied considerably both among provinces and between individual sampling locations within the same province. Increasing latitude was associated with progressively lower odds of overall *Theileria* detection, with each 1° northward increase corresponding to approximately a 22% reduction in odds. This pattern was particularly pronounced for *T. annulata*, whereas the latitudinal association for *T. orientalis* was substantially weaker, indicating that the different *Theileria* taxa do not share a uniform geographic distribution. Latitude may serve as a broad proxy for the north–south climatic and ecological gradient across Kazakhstan, where ixodid tick species show geographically structured distributions and associated differences in pathogen occurrence (4–7). Recent modelling further indicates that climate and land-use changes may modify the suitable habitats of several dominant tick species in Kazakhstan (4, 7). However, because tick abundance, climatic variables, livestock movement, grazing practices, and herd management were not measured or incorporated into the present analysis, the mechanisms underlying the observed latitudinal associations cannot be determined directly. Thus, the observed associations should be interpreted as broad geographic patterns rather than evidence of direct effects of climate, tick abundance, or specific environmental conditions on *Theileria* transmission.

Comparable geographic heterogeneity has also been reported in neighboring countries. In Kyrgyzstan, molecular surveys of cattle identified *T. annulata* and other piroplasm taxa, with variation among sampling regions (22, 23), while *T. orientalis* infection in cattle in Mongolia also showed substantial geographic variation and included five MPSP genotypes (24). In northeastern China, *T. orientalis* and *T. sinensis* were detected in cattle from Yanbian, with differences in detection among sampling locations (25). Co-infections with tick-borne pathogens have also been documented in cattle in Russia (26). Direct comparison of prevalence estimates remains difficult because of differences in sampling design, geographic coverage, host populations, diagnostic methods, and study periods.

The geographic distribution of *Theileria* spp. observed in the present study should also be considered in the context of Kazakhstan’s diverse ixodid tick fauna. Species of *Hyalomma*, *Dermacentor*, *Rhipicephalus*, *Haemaphysalis*, and *Ixodes* occur across different ecological zones of the country, and several species within these genera have been recognized or proposed as vectors of bovine and ovine *Theileria* spp. (4–6). *Theileria annulata* has been associated with *Hyalomma* ticks, whereas transmission of members of the *T. orientalis* complex is generally associated with *Haemaphysalis* spp. (4, 27). These previously reported vector associations provide a possible ecological context for the geographic patterns observed in the present study, but they cannot be directly evaluated here because ticks were not collected concurrently from the sampled animals.

Among the bovine *Theileria* species identified in this study, *T. annulata* showed the highest overall PCR positivity and was detected in 16 of the 17 provinces sampled. Its widespread occurrence indicates that *T. annulata* circulation is not restricted to the historically recognized southern foci of Kazakhstan. Previous molecular studies have demonstrated the circulation of *T. annulata* in southern, western, and eastern parts of the country, although their geographic coverage was substantially more limited (5, 10, 21). The particularly high PCR positivity observed in several southern provinces is also consistent with the long-recognized importance of tropical theileriosis in this part of Kazakhstan (28, 29).

PCR positivity for *T. annulata* in Turkistan and Jambyl provinces differed from estimates reported in earlier studies conducted in these regions (10). Direct comparisons among studies should, however, be interpreted cautiously because of differences in sampling locations, study populations, sampling years, and molecular assays. The same considerations apply to the lower PCR positivity observed in West Kazakhstan Province compared with that reported by Sidikhov et al. (21). These differences highlight the substantial spatial and temporal variation that may affect the detection of tick-borne parasites and further support the need for standardized longitudinal surveillance.

The detection of *T. annulata* in sheep is epidemiologically noteworthy, as the parasite was identified in all five provinces sampled for sheep, including Jetisu, where it was not detected in the sampled cattle. Experimental studies have demonstrated that sheep can sustain *T. annulata* infection, although infection is generally less clinically apparent in sheep than in cattle (30). The presence of *T. annulata* in sheep therefore indicates that parasite circulation is not restricted to cattle, but the contribution of sheep to transmission in Kazakhstan cannot be determined from the present cross-sectional data. *Theileria* infections have also been reported in saiga antelopes (*Saiga tatarica*) in Kazakhstan (31), further indicating that transmission cycles may involve multiple domestic and wild ruminant hosts.

Another notable finding was the frequent co-detection of *T. annulata* and *T. orientalis*. These two species were simultaneously detected in 491 cattle. The odds of *T. orientalis* detection were higher in *T. annulata*-positive cattle (OR = 3.73; 95% CI, 3.23–4.30). Co-detection of tick-borne pathogens has been reported in cattle exposed to overlapping vector populations and have also been reported in other Eurasian settings (26). This association may reflect shared ecological or herd-level exposure rather than direct biological interaction between the two parasites. However, because contemporaneous data on tick infestation, grazing practices, and herd management were unavailable, the factors underlying this association could not be evaluated.

*Theileria orientalis* showed a distribution distinct from that of *T. annulata*. It was detected in all 17 provinces sampled, including several northern and central regions where *T. annulata* PCR positivity was low. Previous studies have also documented *T. orientalis* in Kazakhstan and neighboring Kyrgyzstan, although its reported detection frequency has varied substantially across geographic areas (10, 22, 23). By contrast, a recent study from Kashgar, Xinjiang, reported substantially lower detection of *T. orientalis* than of *T. annulata* (32). These regional differences indicate that the epidemiology of *T. orientalis* in Central Asia is heterogeneous and is unlikely to be explained by a single geographic or ecological factor.

MPSP analysis demonstrated the presence of *T. orientalis* genotypes 1 (“Chitose”), 2 (“Ikeda”), and 3 (“Buffeli”) in Kazakhstan. Because genotyping was performed on a selected subset of *T. orientalis*-positive samples and samples showing mixed 28S rRNA chromatograms were excluded, the observed genotype composition should be interpreted as evidence of genotype occurrence rather than as an estimate of their relative frequencies in the cattle population. Of particular significance was the identification of genotype 2 (“Ikeda”), representing its first molecularly confirmed detection in Kazakhstan. Although genotype 2 is the *T. orientalis* lineage most consistently associated with clinical oriental theileriosis, including anemia, reduced productivity, and mortality (2), its detection in the present study does not by itself establish clinical disease, as all sampled cattle were clinically healthy and clinical outcomes were not assessed. Because ticks were not collected concurrently, the vector species responsible for transmission of genotype 2 in Kazakhstan remains unknown. Its detection supports targeted surveillance of *T. orientalis* genotypes together with contemporaneous characterization of potential tick vectors.

The detection of *T. sinensis* represents another novel finding for Kazakhstan. The species showed low overall PCR positivity and was detected in only five provinces, with the highest values recorded in Pavlodar and North Kazakhstan. Although *T. sinensis* detection was positively associated with latitude in the geographic model, this finding should be interpreted cautiously because only 33 cattle were positive and the analysis did not include climatic, tick-abundance, or other environmental covariates. *T. sinensis* has been reported predominantly from Asia, including China and Russia, with more recent detections extending its documented range westward (26, 32, 33). *Haemaphysalis qinghaiensis* has been implicated as a potential vector in China (33), whereas the vector responsible for transmission in Kazakhstan remains unknown. The low overall PCR positivity and geographically limited detection observed in this study indicate that further targeted sampling is needed to characterize the distribution and veterinary significance of *T. sinensis* in Kazakhstan.

Three *Theileria* taxa were identified in sheep: *T. ovis*, *T. annulata*, and *Theileria* sp. genotype OT3. *Theileria ovis* is generally regarded as a low-pathogenicity parasite of small ruminants, whereas *T. annulata* infections in sheep are usually less clinically apparent than in cattle (1, 30). The clinical significance of *Theileria* sp. genotype OT3 remains poorly defined. In contrast, the major pathogenic ovine *Theileria* species, *T. lestoquardi*, *T. luwenshuni*, and *T. uilenbergi* (34–36), were not detected in the sampled animals. The absence of these species from the sampled animals does not necessarily indicate their absence from Kazakhstan, given that sheep sampling was limited to five southern provinces and clinically healthy adult females.

*Theileria ovis* was the most frequently detected ovine species in this study and was identified in all five sampled provinces, although its overall PCR positivity was 5.5%. Molecular studies from Türkiye and Iran have reported considerable variation in the frequency of *T. ovis* infection, with higher values observed in several settings (37–39). Direct comparisons among studies should be made cautiously because of substantial differences in geographic coverage, host populations, sampling periods, vector exposure, and diagnostic approaches. Earlier microscopy-based investigations in southern Kazakhstan also reported higher occurrence of ovine piroplasm infections (8). The lower PCR positivity observed in this study may reflect differences in study design, sampled populations, and diagnostic methods and should not necessarily be interpreted as evidence of a temporal decline in infection.

The identification of *Theileria* sp. genotype OT3 represents a particularly notable finding of the ovine survey and, to our knowledge, its first molecular detection in Kazakhstan. This genotype has previously been reported in small ruminants from geographically distant regions, including Spain, Iran, Türkiye, and China (38, 40–42). The 18S rRNA sequence obtained from the Kazakhstan isolate was identical to OT3 reference sequences included in our analysis from China and Türkiye, supporting the occurrence of closely related parasite variants across widely separated parts of Eurasia. Recent detection of genotype OT3 in *Dermacentor* ticks in Qinghai, China, provides preliminary evidence of a possible vector association (43). However, the vector responsible for OT3 transmission in Kazakhstan remains unknown. OT3-related sequences have additionally been detected in wild cervids and chamois in Europe (44, 45), suggesting that its host range may extend beyond domestic sheep. Whether wild ruminants contribute to the maintenance of genotype OT3 in Kazakhstan remains to be investigated.

Overall, the ovine survey revealed a species composition distinct from that observed in cattle and showed substantial geographic heterogeneity among the five surveyed provinces. The predominance of *T. ovis* in Kyzylorda and the concentration of genotype OT3 detections in Almaty Province indicate that different *Theileria* taxa may have distinct local transmission patterns. Because concurrent tick collections and information on flock management were unavailable, the ecological determinants of these differences could not be assessed. Broader molecular surveillance incorporating additional regions, age groups, tick sampling, and clinically affected animals will be required to define the epidemiology and clinical relevance of ovine *Theileria* infections in Kazakhstan.

Several limitations should be considered when interpreting the present findings. Sampling was non-probabilistic, depended on voluntary farmer participation, and was not proportional to provincial livestock populations; therefore, the reported PCR positivity estimates characterize the sampled animals and should not be interpreted as population-representative national prevalence estimates. Only clinically healthy females older than three years were included. The age threshold was selected to ensure that animals had experienced at least two grazing/tick-activity seasons and thus sufficient opportunity for natural exposure to *Theileria* spp.; however, younger animals may differ in susceptibility and cumulative exposure, while males may have different exposure patterns and clinically affected animals may have higher parasite loads. Therefore, the findings may not fully reflect *Theileria* infection patterns in these groups or in other seasons. Clinical and hematological outcomes were not assessed, and parasite load was not quantified; therefore, molecular detection cannot be directly related to disease severity or clinical impact. Because sampling was cross-sectional, temporal changes in infection status and parasite dynamics could not be evaluated. Tick infestation and vector competence were not assessed because ticks were not collected, while information on relevant management and exposure factors was unavailable; consequently, potential vector–parasite associations could not be directly evaluated. Co-detection was inferred from bidirectional Sanger sequencing of a genus-level 28S rRNA amplicon and may therefore underestimate low-abundance mixed infections. Such infections may have remained undetected because of the limited sensitivity of Sanger sequencing for minor variants. Next-generation sequencing approaches could provide deeper resolution of mixed *Theileria* populations and improve detection of low-abundance co-infecting taxa. The analytical sensitivity of the 28S rRNA PCR was not compared with other commonly used *Theileria* markers; therefore, its relative sensitivity cannot be inferred. Finally, MPSP genotyping was performed on a selected subset of *T. orientalis*-positive samples; consequently, the identified genotypes demonstrate their occurrence in Kazakhstan but should not be interpreted as estimates of genotype frequency in the cattle population.

## 5 Conclusion

This study provides a geographically extensive molecular characterization of *Theileria* spp. in cattle across Kazakhstan and of ovine infections in five southern provinces. *Theileria annulata* and *T. orientalis* were widely detected in cattle, but their occurrence showed marked spatial heterogeneity, whereas *T. sinensis* was detected at low frequency in five provinces. The identification of *T. orientalis* genotype 2 (“Ikeda”), *T. sinensis*, and *Theileria* sp. genotype OT3 expands the known diversity of *Theileria* circulating in Kazakhstan. Frequent co-detection of *T. annulata and T. orientalis* further indicates shared epidemiological exposure within the sampled cattle population. These findings provide a baseline for geographically targeted molecular surveillance integrating parasite genotyping, systematic tick monitoring, and epidemiologically relevant information on livestock movement and management.

## Data Availability

All data from this project is publicly available on NCBI, GenBank accession numbers: PZ312048–PZ312062, PZ312064–PZ312066, and PZ319530–PZ319536. Sample identifiers, target genes, and corresponding accession numbers are provided in Supplementary Table S3.

## Supplementary material

SUPPLEMENTARY TABLE S1 Sampled settlements, geographic coordinates, PCR positivity for Theileria spp. in adult cattle, and provincial cattle population sizes.

SUPPLEMENTARY TABLE S2 Sampled settlements, geographic coordinates, PCR positivity for Theileria spp. in female sheep, and provincial sheep population sizes.

SUPPLEMENTARY TABLE S3 GenBank accession numbers, sample identifiers, and target genes of Theileria sequences generated in this study.

SUPPLEMENTARY TABLE S4 Geographic associations between latitude and PCR detection of Theileria spp. in cattle based on generalized estimating equations.

SUPPLEMENTARY TABLE S5 Reference Theileria and Babesia sequences used for phylogenetic analysis, including GenBank accession numbers, gene markers, hosts, and countries of origin.
