# Supplementary material for: Molecular epidemiology and geographic distribution of Theileria spp. in Kazakhstan: a countrywide cattle survey and a southern sheep survey
Source: Front Vet Sci. 2026 Oct 7;13:1987322. doi: 10.3389/fvets.2026.1987322 (PMC13683191; doi:10.3389/fvets.2026.1987322)
Supplement: Supplementary file 1 [file Data_Sheet_1.PDF]

Supplementary Table S1. Sampled settlements, geographic coordinates (from Google Maps), PCR positivity for Theileria spp. in adult cattle (cows) from 172 settlements across 17 provinces of Kazakhstan (sampling conducted in July–August 2022 and 2023), and provincial cattle population sizes at the end of 2023 (http://stat.gov.kz).

Province (oblast)	Administrative district	Settlement	Latitude	Longitude	N tested using PCR	*Theileria* spp.		*T. annulata*		*T. orientalis*		*T. sinensis*		Cattle (cow) populationa
						N pos.	% pos.b	N pos.	% pos.b	N pos.	% pos.b	N pos.	% pos.b	
**Abai**	**Subtotal province**				**382**	**47**	12.3 (9.2–16.0)	**27**	7.1 (4.7–10.1)	**30**	7.9 (5.4–11.0)	**0**	0 (0–1.0)	**697,000 (408,000)**
	Ayagoz	Ayagoz	47,965266	80,436238	48	7	15	7	15	0	0	0	0	
	Beskaragay	Beskarahay	50,886001	79,480996	48	12	25	11	23	4	8	0	0	
	Kurchatov	Kurchatov	50,756325	78,548329	41	0	0	0	0	0	0	0	0	
	Zharma	Qalbatau	49,328084	81,573693	49	0	0	0	0	0	0	0	0	
	Abay	Kengirbay bi	48,902261	79,246401	49	2	4	0	0	2	4	0	0	
	Kokpekti	Ulken Boken	48,781692	82,694252	50	17	34	0	0	17	34	0	0	
	Borodulikha	Mikhaylichenkovo	50,643906	81,135253	50	0	0	0	0	0	0	0	0	
	Zhanasemey	Dostyk	50,454444	79,851389	47	9	19	9	19	7	15	0	0	
**Akmola**	**Subtotal province**				**179**	**21**	11.7 (7.4–17.4)	**12**	6.7 (3.5–11.4)	**18**	10.1 (6.1–15.4)	**0**	0 (0–2.0)	**453,000 (233,000)**
	Arshaly	Saryoba	51,203207	72,183154	179	21	12,0	12	7	18	10	0	0	
**Aktobe**	**Subtotal province**				**175**	**3**	1.7 (0.4–4.9)	**1**	0.6 (0.01–3.1)	**2**	1.1 (0.1–4.1)	**0**	0 (0–2.1)	**669,000 (367,000)**
	Yrgyz	Zhaysanbai	48,039595	62,512135	47	0	0	0	0	0	0	0	0	
	Bayganin	Zharly	48,532598	55,591136	47	0	0	0	0	0	0	0	0	
	Temir	Jaksimaj rural district	49,203889	56,562500	46	1	2	1	2	0	0	0	0	
	Oiyl	Oiyl	49,074441	54,665629	35	2	6	0	0	2	6	0	0	
**Almaty**	**Subtotal province**				**234**	**125**	53.4 (46.8–59.9)	**40**	17.1 (12.5–22.5)	**103**	44.0 (37.6–50.6)	**0**	0 (0–1.6)	**657,000 (412,000)**
	Enbekshikazakh	Karashota	43,649992	78,183065	20	13	65	12	60	10	50	0	0	
		Asa rural district	43,526944	78,276111	10	8	80	8	80	2	20	0	0	
		Shelek rural district	43,599167	78,256667	8	5	63	5	63	0	0	0	0	

…continued.

Province (oblast)	Administrative district	Settlement	Latitude	Longitude	N tested using PCR	*Theileria* spp.		*T. annulata*		*T. orientalis*		*T. sinensis*		Cattle (cow) populationa
						N pos.	% pos.b	N pos.	% pos.b	N pos.	% pos.b	N pos.	% pos.b	
Almaty	Talgar	Nura	43,687611	77,193041	46	26	57	1	2	26	57	0	0	
	Zhambyl	Uzynagash	43,220925	76,314210	42	23	55	10	24	18	43	0	0	
		Sholak	44,037222	77,774722	33	8	24	1	3	8	24	0	0	
	Ile	Mezhdurechenskoye	43,455160	76,723780	6	5	83	1	17	4	67	0	0	
		Akshi	43,991220	76,326822	26	13	50	0	0	13	50	0	0	
		Zhetygen	43,681261	77,116046	11	9	82	0	0	9	82	0	0	
		Karaoy	43,517111	76,833212	16	13	81	0	0	13	81	0	0	
		Baiserke	43,489634	77,040615	16	2	13	2	13	0	0	0	0	
Atyrau	**Subtotal province**				**194**	**28**	14.4 (9.8–20.2)	**27**	13.9 (9.4–19.6)	**2**	1.0 (0.1–3.7)	**0**	0 (0–1.9)	**204,000 (118,000)**
	Zhylyoi	Kulsary	46,960532	54,006221	22	1	5	0	0	1	5	0	0	
		Aqqyztoghay	47,096629	54,375887	24	0	0	0	0	0	0	0	0	
	Inder	Inderbor	48,555353	51,742215	24	0	0	0	0	0	0	0	0	
		Koktogay	48,131198	51,534873	24	0	0	0	0	0	0	0	0	
	Isatay	Tushchikuduk	47,260314	51,067948	25	0	0	0	0	0	0	0	0	
		Rybachje	47.685336	50.839022	25	8	32	8	32	0	0	0	0	
	Kurmangazy	Primorye	46,550121	49,209460	50	19	38	19	38	1	2	0	0	
East Kazakhstan	**Subtotal province**				**197**	**30**	15.2 (10.5–21.0)	**2**	1.0 (0.1–3.6)	**30**	15.2 (10.5–21.0)	**0**	0 (0–1.9)	**440,000 (246,000)**
	Ulan	Aiyrtau	49,836250	82,265001	50	2	4	0	0	2	4	0	0	
		Gagarino	50,144975	81,848838	49	20	41	0	0	20	41	0	0	
	Glubokoye	Solnechnoe	50,050572	82,714572	98	8	8	2	2	8	8	0	0	
Jambyl	**Subtotal province**				**851**	**527**	61.9 (58.6–65.2)	**396**	46.5 (43.1–50.0)	**258**	30.3 (27.2–33.5)	**1**	0.1 (0.003–0.7)	**530,000 (228,000)**
	Bayzak	Botamoynak rural district	42,978056	71,527500	47	47	100	47	100	47	100	0	0	
		Bayzak	42,977381	71,529450	50	44	88	44	88	37	74	0	0	
		Akchulak	43,005541	71,802885	26	19	73	19	73	4	15	0	0	

…continued.

Province (oblast)	Administrative district	Settlement	Latitude	Longitude	N tested using PCR	*Theileria* spp.		*T. annulata*		*T. orientalis*		*T. sinensis*		Cattle (cow) populationa
						**N pos.**	**% pos.b**	**N pos.**	**% pos.b**	**N pos.**	**% pos.b**	**N pos.**	**% pos.b**	
**Jambyl**	Bayzak	Kolkaynar	43,059167	71,235556	47	47	100	47	100	5	11	0	0	
		Karatobinskoye	42,883057	71,284225	28	24	86	22	79	22	79	0	0	
	Turar Ryskulova	Terenonzek	42,978694	72,066562	50	49	98	49	98	0	0	0	0	
		Abay rural district	42,912313	72,800952	50	44	88	0	0	44	88	0	0	
		Akyrtobe	42,991073	72,101704	49	43	88	4	8	39	80	0	0	
	Talas	Berikkainar rural district	43,012481	70,580326	47	27	57	26	55	1	2	0	0	
		Koktal	43,266107	70,301813	47	35	74	32	68	3	6	0	0	
		Karatau	43,175515	70,459523	28	0	0	0	0	0	0	0	0	
	Zhualy	Shinbulak	42,706488	70,650511	47	0	0	0	0	0	0	0	0	
		Kolbastau	42,607500	70,784167	48	29	60	29	60	1	2	0	0	
		Aksay	42,604679	70,762693	48	39	81	39	81	4	8	0	0	
		Bauyrjan Momyshuly rural district	42,619051	70,764328	49	40	82	3	6	40	82	0	0	
	Merki	Merki	42,872481	73,190139	190	40	21	35	18	11	6	1	1	
**Jetisu**	**Subtotal province**				**200**	**72**	36.0 (29.4–43.1)	**0**	0 (0–1.8)	**72**	36.0 (29.4–43.1)	**0**	0 (0–1.8)	**516,000 (275,000)**
	Koksu	Jarlyozek	44,904599	78,054974	50	18	36	0	0	18	36	0	0	
	Aksu	Zhansugiruly	45,398587	79,503265	50	6	12	0	0	6	12	0	0	
	Eskeldi	Syrymbet	44,856519	78,682484	50	0	0	0	0	0	0	0	0	
	Kerbulak	Zhaynak	44,388171	78,585834	50	48	96	0	0	48	96	0	0	
**Karaganda**	**Subtotal province**				**496**	**2**	0.4 (0.05–1.4)	**2**	0.4 (0.05–1.4)	**1**	0.2 (0.005–1.1)	**0**	0 (0–0.7)	**486,000 (267,000)**
	Nura	Ahmet	50,635851	71,462418	49	1	2	1	2	1	2	0	0	
		Karaoy	50,256022	71,483187	50	0	0	0	0	0	0	0	0	
	Priozersk	Priozersk	46,028969	73,708027	50	0	0	0	0	0	0	0	0	
	Sarybel	Oshagandy	50,312947	72,734900	20	0	0	0	0	0	0	0	0	

…continued.

Province (oblast)	Administrative district	Settlement	Latitude	Longitude	N tested using PCR	*Theileria* spp.		*T. annulata*		*T. orientalis*		*T. sinensis*		Cattle (cow) population[a]
						N pos.	% pos.[b]	N pos.	% pos.[b]	N pos.	% pos.[b]	N pos.	% pos.[b]	
Karaganda	Sarybel	Molodezhny y	50,726109	73,529028	15	0	0	0	0	0	0	0	0	
		Sunkar	50,381638	72,220192	15	0	0	0	0	0	0	0	0	
	Shet	Kayrakty	48,849579	73,603831	20	0	0	0	0	0	0	0	0	
		Kenshoki rural district	48,574444	73,983611	19	0	0	0	0	0	0	0	0	
		Akadyr	48,263632	72,856047	20	0	0	0	0	0	0	0	0	
		Akoi	49,021557	72,560097	20	0	0	0	0	0	0	0	0	
		Taldy	49,107591	73,748038	20	0	0	0	0	0	0	0	0	
	Karkaraly	Karakezen	49,595000	75,058333	49	1	2	1	2	0	0	0	0	
	Abai	Kulajgyr	49,425908	72,923348	50	0	0	0	0	0	0	0	0	
	Balkhash	Balkhash	46,843721	74,977301	20	0	0	0	0	0	0	0	0	
		Sayak	46,906506	77,356417	19	0	0	0	0	0	0	0	0	
		Gulshat	46,630004	74,355452	11	0	0	0	0	0	0	0	0	
	Aktogay	Nurken	48,107764	74,561076	7	0	0	0	0	0	0	0	0	
		Karamende Bi rural district	48,630000	75,028333	5	0	0	0	0	0	0	0	0	
		Zhidebay	47,811234	74,773941	9	0	0	0	0	0	0	0	0	
		Shabanbai Bi	48,402289	75,394254	8	0	0	0	0	0	0	0	0	
		Aktogay	48,316087	74,981362	8	0	0	0	0	0	0	0	0	
		Kyzylaray rural district	48,572778	75,487222	8	0	0	0	0	0	0	0	0	
		Saryterek	48,068076	75,338720	4	0	0	0	0	0	0	0	0	
	**Subtotal province**				**363**	**39**	10.7 (7.8–14.4)	**37**	10.2 (7.3–13.8)	**36**	9.9 (7.0–13.5)	**1**	0.3 (0.007–1.5)	**477,000 (249,000)**
Kostanay	Denisov	Pereleski	52,545399	62,074871	50	1	2	1	2	1	2	0	0	
	Kamysty	Kamysty	51,963814	61,779038	48	5	10	5	10	5	10	0	0	
	Karabalyk	Nadezhdinka	53,760294	63,743249	38	6	16	6	16	6	16	0	0	

…continued.

Province (oblast)	Administrative district	Settlement	Latitude	Longitude	N tested using PCR	*Theileria* spp.		*T. annulata*		*T. orientalis*		*T. sinensis*		Cattle (cow) population[a]
						N pos.	% pos.[b]	N pos.	% pos.[b]	N pos.	% pos.[b]	N pos.	% pos.[b]	
**Kostanay**	Fyodorov	Fyodorovka	53,636244	62,698023	40	14	35	14	35	14	35	1	3	
	Altynsarin	Tanabay	53,345962	64,578359	40	2	5	2	5	2	5	0	0	
	Amangeldi	Amangeldy	50,183089	65,186320	33	1	3	1	3	1	3	0	0	
	Zhangeldi	Torghay	49,632447	63,491837	38	4	11	4	11	4	11	0	0	
	Sarykol	Ostrovnoye	53,342672	65,477797	48	6	13	4	8	3	6	0	0	
	Kostanay	Zarechnyi	53.2337054	63.736094	28	0	0	0	0	0	0	0	0	
**Kyzylorda**	**Subtotal province**				**848**	**499**	58.8 (55.4–62.2)	**499**	58.8 (55.4–62.2)	**136**	16.0 (13.6–18.7)	**0**	0 (0–0.4)	**410,000 (243,000)**
	Karmakshy	3-d Internatsional	45,191667	64,128889	47	34	72	34	72	0	0	0	0	
		Taimbet Komekbaev rural district	44,838933	63,197639	52	27	52	27	52	1	2	0	0	
	Kyzylorda	Kyzylorda	44,842544	65,502563	50	17	34	17	34	0	0	0	0	
	Aral	Aral	46,797738	61,660792	50	20	40	20	40	0	0	0	0	
		Zhaksylysh rural district	46,915489	62,026956	26	4	15	4	15	1	4	0	0	
	Aral	Zhaksykylysh	46,824752	61,854119	25	4	16	4	16	0	0	0	0	
		Akkum rural district	46,942778	61,620000	25	9	36	9	36	0	0	0	0	
	Zhalagash	Zhalagash	45,080281	64,681333	42	5	12	5	12	1	2	0	0	
		Akkum	44,916664	64,712612	46	12	26	12	26	0	0	0	0	
	Kazaly	Ayteke Bi	45,835934	62,148317	31	13	42	13	42	0	0	0	0	
		Sarykol rural district	45,732557	62,115942	47	44	94	44	94	3	6	0	0	

…continued.

Province (oblast)	Administrative district	Settlement	Latitude	Longitude	N tested using PCR	*Theileria* spp.		*T. annulata*		*T. orientalis*		*T. sinensis*		Cattle (cow) populationa
						N pos.	% pos.b	N pos.	% pos.b	N pos.	% pos.b	N pos.	% pos.b	
**Kyzylorda**	Zhanakorgan	Zhanakorgan	43,904444	67,251111	47	35	74	35	74	4	9	0	0	
**Kyzylorda**	Zhanakorgan	Zhamanbai batyr rural district	43,689722	67,766111	37	36	97	36	97	36	97	0	0	
		Aqqorghan rural district	43,592500	67,411111	45	21	47	21	47	0	0	0	0	
		Kirkense rural district	43,798333	67,124722	48	27	56	27	56	1	2	0	0	
	Shieli	Telekol rural district	44,259722	66,685556	49	45	92	45	92	9	18	0	0	
		Zhuantobe	44,267588	66,311218	43	39	91	39	91	9	21	0	0	
		Shieli	44,178889	66,732778	47	32	68	32	68	13	28	0	0	
	Syrdariya	Terenozek	45,051944	64,980556	45	33	73	33	73	24	53	0	0	
		Askar Tekmagambetov rural district	45,057148	65,052480	46	42	91	42	91	34	74	0	0	
**Mangystau**	**Subtotal province**				**180**	**54**	30.0 (23.4–37.3)	**54**	30.0 (23.4–37.3)	**1**	0.6 (0.01–3.1)	**0**	0 (0–2.0)	**21,000 (15,000)**
	Mangystau	Say-Otes	44,328530	53,533572	15	8	53	8	53	0	0	0	0	
		Shetpe	44,139155	52,162069	12	12	100	12	100	0	0	0	0	
		Zhyngyldy	44,198686	51,700201	8	7	88	7	88	0	0	0	0	
		Otpan rural district	44,149809	51,942377	4	3	75	3	75	0	0	0	0	
	Beyneu	Sam	45,236993	55,866650	11	4	36	4	36	0	0	0	0	
		Beyneu	45,322946	55,188862	15	2	13	2	13	1	7	0	0	
		Boranqul	46,208008	54,469042	13	4	31	4	31	0	0	0	0	
	Karakiya	Akjigit	45,039267	55,753112	7	1	14	1	14	0	0	0	0	
		Kuryk	43,178221	51,679423	21	4	19	4	19	0	0	0	0	
		Zhetybay	43,587320	52,104326	24	2	8	2	8	0	0	0	0	

…continued.

Province (oblast)	Administrative district	Settlement	Latitude	Longitude	N tested using PCR	*Theileria* spp.		*T. annulata*		*T. orientalis*		*T. sinensis*		Cattle (cow) populationa
						N pos.	% pos.b	N pos.	% pos.b	N pos.	% pos.b	N pos.	% pos.b	
**Mangystau**	Munaily	Daulet	43,701879	51,321067	10	2	20	2	20	0	0	0	0	
	Munaily	Mangystau	43,691561	51,305571	10	0	0	0	0	0	0	0	0	
		Kyzyltobe	43,692930	51,337874	10	0	0	0	0	0	0	0	0	
		Batyr	43,630290	51,408113	10	2	20	2	20	0	0	0	0	
		Bayandy	43,761104	51,306083	10	3	30	3	30	0	0	0	0	
**North Kazakhstan**	**Subtotal province**				**320**	**108**	33.8 (28.6–39.2)	**19**	5.9 (3.6–9.1)	**99**	30.9 (25.9–36.3)	**8**	2.5 (1.1–4.9)	**380,000 (224,000)**
	Zhambyl	Presnovka	54,671539	67,158203	48	26	54	2	4	26	54	1	2	
	Mamlyut	Shchuchye	55,153509	68,506134	9	3	33	1	11	3	33	0	0	
		Beloe	55,062823	68,473939	9	1	11	0	0	1	11	0	0	
		Krasnoznamennoe	54,977356	68,808948	9	7	78	1	11	0	0	6	67	
		Pokrovka	54,928831	68,636794	19	5	26	0	0	5	26	0	0	
		Ledenevo	54,699696	68,535069	20	6	30	2	10	5	25	0	0	
	Akkayin	Vlasovka	54,200965	69,600741	38	31	82	10	26	31	82	0	0	
		Kuchkova	54,146761	69,783296	4	0	0	0	0	0	0	0	0	
	Kyzylzhar	Semicol	54,600781	68,827183	35	16	46	2	6	16	46	1	3	
	Magzhan Zhumabaev	Zhastar	54,566944	70,212222	21	8	38	0	0	8	38	0	0	
		Sortomar	54,638351	70,167560	20	2	10	0	0	2	10	0	0	
	Esil	Amangeldy	54,177274	67,937977	61	0	0	0	0	0	0	0	0	
		Ilinka	54,201824	68,010444	10	1	10	0	0	1	10	0	0	
	Timiryazev	Dokuchaevo	53,914759	66,486964	15	2	13	1	7	1	7	0	0	
	Taiynsha	Karagash	53,630269	70,119024	2	0	0	0	0	0	0	0	0	
**Pavlodar**	**Subtotal province**				**260**	**21**	8.1 (5.1–12.1)	**5**	1.9 (0.6–4.4)	**21**	8.1 (5.1–12.1)	**19**	7.3 (4.5–11.2)	**480,000 (275,000)**
	Zhelezin	Tserkovnoye	53,603723	75,915951	45	0	0	0	0	0	0	0	0	
	Sharbaqty	Orlovka	52,576182	78,032966	23	1	4	1	4	1	4	0	0	
	Sharbaqty	Krasilovka	52,712993	77,792648	17	3	18	0	0	3	18	3	18	
	Akkuly	Caracala	51,722183	77,533026	47	12	26	0	0	12	26	12	26	

…continued.

Province (oblast)	Administrative district	Settlement	Latitude	Longitude	N tested using PCR	*Theileria* spp.		*T. annulata*		*T. orientalis*		*T. sinensis*		Cattle (cow) population[a]
						**N pos.**	**% pos.[b]**	**N pos.**	**% pos.[b]**	**N pos.**	**% pos.[b]**	**N pos.**	**% pos.[b]**	
**Pavlodar**	Uspen	Uspenka	52,907129	77,419380	45	4	9	4	9	4	9	4	9	
	Ertis	Kosagash	53,114579	74,870959	43	0	0	0	0	0	0	0	0	
	Pavlodar	Pavlodar	52,285577	76,940722	40	1	3	0	0	1	3	0	0	
**Turkistan**	**Subtotal province**				**719**	**477**	66.3 (62.8–69.8)	**477**	66.3 (62.8–69.8)	**128**	17.8 (15.1–20.8)	**4**	0.6 (0.2–1.4)	**1,083,000 (639,000)**
	Sayram	Karabulak	42,533632	69,755351	40	0	0	0	0	0	0	0	0	
		Karamurt	42,307077	69,969777	20	0	0	0	0	0	0	0	0	
		Mankent	42,440391	69,874677	20	0	0	0	0	0	0	0	0	
	Badam rural district	Badam settlement	42,381820	69,256509	40	0	0	0	0	0	0	0	0	
	Borjar	Uyalyzhar	42,431613	69,294784	40	0	0	0	0	0	0	0	0	
	Otrar	Aktobe	42,799580	68,713191	33	4	12	4	12	0	0	0	0	
		Talapty	42,861180	68,294127	40	39	98	39	98	29	73	0	0	
	Tulkibas	Abay	42,641091	70,114274	88	80	91	80	91	34	39	0	0	
	Kazygurt	Sharbulak	41,774444	69,490278	50	43	86	43	86	17	34	0	0	
	Tole Bi	Zhogargi Aksu	42,331369	70,067498	58	50	86	50	86	9	16	4	7	
	Sauran	Karashyk	43,357736	68,231624	43	43	100	43	100	1	2	0	0	
		Chernak	43,399573	68,016379	48	48	100	48	100	13	27	0	0	
	Saryagash	Han-Zhan	41,599341	69,347308	32	25	78	25	78	2	6	0	0	
	Baydibek	Kenestobe	43,036235	69,610353	46	46	100	46	100	0	0	0	0	
	Sozak	Syzgan	43,978105	68,545822	49	49	100	49	100	1	2	0	0	
		Sozak	44,138605	68,467201	24	24	100	24	100	18	75	0	0	
	Ordabasy	Temirlan	42,599137	69,256309	48	26	54	26	54	4	8	0	0	
**Ulytau**	**Subtotal province**				**277**	**61**	22.0 (17.3–27.4)	**61**	22.0 (17.3–27.4)	**3**	1.1 (0.2–3.1)	**0**	0 (0–1.3)	**149,000 (107,000)**
	Ulytau	Mibulak	46,949910	68,287377	49	31	63	31	63	0	0	0	0	
	Zhezqazghan	Kengir	47,836945	67,618815	45	2	4	2	4	0	0	0	0	
	Zhezqazghan	Zhezqazghan	47,799711	67,714090	50	0	0	0	0	0	0	0	0	

…continued.

Province (oblast)	Administrative district	Settlement	Latitude	Longitude	N tested using PCR	*Theileria* spp.		*T. annulata*		*T. orientalis*		*T. sinensis*		Cattle (cow) populationa
						N pos.	% pos.b	N pos.	% pos.b	N pos.	% pos.b	N pos.	% pos.b	
**Ulytau**	Zhanaarka	Saken Seifullin rural district	48,903333	71,753333	47	13	28	13	28	2	4	0	0	
		Bidaik	48,725556	71,943889	36	13	36	13	36	1	3	0	0	
		Zhanaarka	48,685790	71,635155	50	2	4	2	4	0	0	0	0	
	**Subtotal province**				**349**	**28**	8.0 (5.4–11.4)	**26**	7.4 (4.9–10.7)	**2**	0.6 (0.07–2.1)	**0**	0 (0–1.1)	**857,000 (426,000)**
**West Kazakhstan**	Bokey Orda	Karakol	48,416667	46,616667	41	1	2	0	0	1	2	0	0	
	Zhanakala	Zhanazholsky	48,863829	49,892862	43	1	2	0	0	1	2	0	0	
		Zhanaqala	49,212598	50,298241	53	0	0	0	0	0	0	0	0	
	Akzhaik	Zhanabulak	50,314926	51,068254	94	4	4	4	4	0	0	0	0	
	Taskala	Togaily	50,915467	50,550671	71	22	31	22	31	0	0	0	0	
	Kaztal	Bostandyk	49,624068	48,881197	47	0	0	0	0	0	0	0	0	
**Total**					**6 224**	**2 142**	34.4 (33.2–35.6)	**1 685**	27.1 (26.0–28.2)	**942**	15.1 (14.3–16.0)	**33**	0.5 (0.4–0.7)	**8,609,000 (4,766,000)**

igures rounded.

s (Clopper–Pearson) are shown in parentheses.
