# Supplementary material for: Molecular epidemiology and geographic distribution of Theileria spp. in Kazakhstan: a countrywide cattle survey and a southern sheep survey
Source: Front Vet Sci. 2026 Oct 7;13:1987322. doi: 10.3389/fvets.2026.1987322 (PMC13683191; doi:10.3389/fvets.2026.1987322)
Supplement: Supplementary file 2 [file Data_Sheet_2.PDF]

Supplementary Table S2. Sampled settlements, geographic coordinates (from Google Maps), PCR positivity for *Theileria* spp. inadult female sheep (ewes) from 52 settlements across five southern provinces of Kazakhstan (sampling conducted in July–August 2022 and 2023), and provincial cattle population sizes at the end of 2023 (http://stat.gov.kz).

Province (oblast)	Administrative district	Settlement	Latitude	Longitude	N tested using PCR	Theileria spp.		T. ovis		T. annulata		Genotype OT3		Sheep population[a]
						N pos.	% pos.[b]	N pos.	% pos.[b]	N pos.	% pos.[b]	N pos.	% pos.[b]	
	**Subtotal province**				**291**	**65**	**22.3(17.7–27.6)**	**2**	**0.7(0.08–2.5)**	**1**	**0.3(0.009–1.9)**	**63**	**21.6(17.1–26.8)**	**2,340,000**
	Enbekshikazak	Karashota	43,649992	78,183065	17	11	65							
	Talgar	Nura	43,687611	77,193041	97	10	10							
	Zhambyl	Mynbaev	43,294611	76,450790	29	0	0							
**Almaty**	Ile	Zhetygen	43,681261	77,116046	14	2	14							
	Ile	Karaoy	43,517111	76,833212	19	1	5							
	Ile	Baiserke	43,489634	77,040615	18	0	0							
	Karasay	Karatobe	43,424313	76,731262	48	41	85							
	Karasay	Koktogan	43,328223	76,764203	49	0	0							
	**Subtotal province**				**280**	**3**	**1.1(0.2–3.1)**	**3**	**1.1(0.2–3.1)**	**2**	**0.7(0.09–2.6)**	**0**	**0(0–1.3)**	**3,420,000**
	Bayzak	Kokbastau	43,057866	71,643991	30	0	0							
	Bayzak	Bayzak	42,977381	71,529450	56	3	5							
	Jambyl	Ornek	43,083320	70,878926	30	0	0							
	Jambyl	Karatobe	42,883057	71,284225	23	0	0							
	Jambyl	Ernazar	43,132980	70,839940	10	0	0							
**Jambyl**	Turar Ryskulo	Abay rural dist	42,912313	72,800952	22	0	0							
	Talas	Berikkainar	43,012481	70,580326	23	0	0							
	Talas	Karatau	43,175515	70,459523	22	0	0							
	Zhualy	Shinbulak	42,706488	70,650511	24	0	0							
	Zhualy	Kolbastau	42,607500	70,784167	27	0	0							
	Zhualy	Aksay	42,604679	70,762693	13	0	0							
	**Subtotal province**				**296**	**3**	**1.0(0.2–2.9)**	**1**	**0.3(0.009–1.9)**	**3**	**1.0(0.2–2.9)**	**2**	**0.7(0.08–2.4)**	**1,488,000**
	Aksu	Esbolatov	45,412154	79,587680	50	0	0							
	Eskeldi	Karabulak	44,909355	78,489031	46	2	4							
	Kerbulak	Altynemel	44,333643	78,451634	50	0	0							
**Jetisu**	Karatal	Kishitobe	45,345439	77,929291	20	0	0							
	Karatal	Kalpe	45,090947	77,907255	10	0	0							
	Karatal	Kokpekty	45,275010	77,884968	20	0	0							
	Koksu	Mukry	44,859765	78,171737	50	0	0							
	Taldykorgan	Utenay	45,006769	78,318657	50	1	2							
	**Subtotal province**				**385**	**87**	**22.6(18.5–27.1)**	**64**	**16.6(13.0–20.7)**	**28**	**7.3(4.9–10.3)**	**0**	**0(0–1.0)**	**675,000**
**Kyzylorda**	Karmakshy	3-d Internatsio	45,191667	64,128889	19	2	11							
	Kyzylorda	Kyzylorda	44,842544	65,502563	22	4	18							
	Kyzylorda	Kosshynyrau r	44,947222	65,544167	28	2	7							

…continued.

Province (oblast)	Administrative district	Settlement	Latitude	Longitude	N tested using PCR	Theileria spp.		T. ovis		T. annulata		Genotype OT3		Sheep population[a]
						N pos.	% pos.[b]	N pos.	% pos.[b]	N pos.	% pos.[b]	N pos.	% pos.[b]	
Kyzylorda	Aral	Zhaksykylysh	46,824752	61,854119	15	0	0							
	Kazaly	Ayteke Bi	45,835934	62,148317	28	0	0							
	Kazaly	Sarykol rural d	45,732557	62,115942	25	2	8							
	Zhalagash	Zhalagash	45,080281	64,681333	30	0	0							
	Zhalagash	Akkum	44,916664	64,712612	14	0	0							
	Zhalagash	Akkorgan	43,592500	67,411111	28	15	54							
	Zhalagash	Kirkense	43,592500	67,411111	27	27	100							
	Shieli	Telekol rural d	44,259722	66,685556	30	0	0							
	Shieli	Zhuantobe	44,267588	66,311218	27	6	22							
	Shieli	Shieli	44,178889	66,732778	5	0	0							
	Shieli	Kerdeli	44,192500	66,781944	33	14	42							
	Syrdariya	Terenozek	45,051944	64,980556	29	12	41							
	Syrdariya	Askar Tekmag	45,057148	65,052480	25	3	12							
	**Subtotal province**				**215**	**11**	**5.1(2.6–9.0)**	**10**	**4.7(2.3–8.4)**	**4**	**1.9(0.5–4.7)**	**0**	**0(0–1.7)**	**4,394,000**
Turkistan	Sayram	Karasu	42,405962	69,795713	13	3	23							
	Ordabasy	Burdjar	43,296298	68,207760	11	0	0							
	Tulkibas	Abay	42,641091	70,114274	47	0	0							
	Kazygurt	Sharbulak	41,774444	69,490278	30	0	0							
	Sauran	Karashyk	43,357736	68,231624	20	4	20							
	Sauran	Chernak	43,399573	68,016379	25	0	0							
	Saryagash	Zhilga	41,718253	69,013000	19	4	21							
	Saryagash	Akzhar	41,598945	69,347344	23	0	0							
	Baydibek	Kenestobe	43,036235	69,610353	27	0	0							
**Total in 5 southern provinces**					**1467**	**169**	**11.5(9.9–13.3)**	**80**	**5.5(4.3–6.7)**	38	2.6(1.8–3.5)	**65**	**4.4(3.4–5.6)**	**12,317,000**

[a] Figures rounded.

[b] Exact 95% confidence intervals (Clopper–Pearson) are shown in parentheses.
