# Supplementary material for: Molecular epidemiology and geographic distribution of Theileria spp. in Kazakhstan: a countrywide cattle survey and a southern sheep survey
Source: Front Vet Sci. 2026 Oct 7;13:1987322. doi: 10.3389/fvets.2026.1987322 (PMC13683191; doi:10.3389/fvets.2026.1987322)
Supplement: Supplementary file 3 [file Data_Sheet_3.PDF]

Supplementary Table S3. GenBank accession numbers of *Theileria* sequences generated in this study, including sample identifiers and target genes.

Sample ID	Host species	Isolation location	Species	Gene	GenBank accession number
NORTHK-5497	Cattle	North Kazakhstan Region, Mamlyut District, Pokrovka	Theileria orientalis isolate Chitose	MPSP	PZ319530
JMB-3070	Cattle	Zhambyl Region, Zhualy District, Bauyrzhan Momyshuly rural district	Theileria orientalis genotype 5	MPSP	PZ319531
ABAY-7272	Cattle	Abai Region, Kokpekti District, Ulken Boken	Theileria orientalis genotype 5	MPSP	PZ319532
ALM-8044	Cattle	Almaty Region, Ile District, Zhetygen	Theileria orientalis genotype 5	MPSP	PZ319533
EASTK-9246	Cattle	East Kazakhstan Region, Ulan District, Gagarino	Theileria orientalis genotype 5	MPSP	PZ319534
NORTHK-5438	Cattle	North Kazakhstan Region, Zhambyl District, Presnovka	*Theileria orientalis* genotype 1 (Chitose)	MPSP	PZ319535
ALM-7607	Cattle	Almaty Region, Enbekshikazakh District, Karashota	*Theileria orientalis* genotype 2 (Ikeda)	MPSP	PZ319536
TRK-714	Cattle	Turkistan Region, Kazygurt District, Sharbulak	*Theileria annulata*	28S rRNA	PZ312048
ULT-6923	Cattle	Ulytau Region, Zhanaarka District, Zhanaarka	*Theileria annulata*	28S rRNA	PZ312049
ULT-6764	Cattle	Ulytau Region, Zhezkazghan District, Kengir	*Theileria annulata*	28S rRNA	PZ312050
ULT-6900	Cattle	Ulytau Region, Zhanaarka District, Bidaik	*Theileria annulata*	28S rRNA	PZ312051
ABAY-7080	Cattle	Abai Region, Beskaragay District, Beskarahay	*Theileria annulata*	28S rRNA	PZ312052
NORTHK-9911	Cattle	North Kazakhstan Region, Timiryazev District, Dokuchaevo	*Theileria annulata*	28S rRNA	PZ312053
NORTHK-5490	Cattle	North Kazakhstan Region, Mamlyut District, Krasnoznamennoe	*Theileria sinensis*	28S rRNA	PZ312054
ALM-7964	Cattle	Almaty Region, Talgar District, Nura	*Theileria orientalis*	28S rRNA	PZ312055
NORTHK-9985	Cattle	North Kazakhstan Region, Mamlyut District, Ledenevo	*Theileria orientalis*	28S rRNA	PZ312056
NORTHK-5538	Cattle	North Kazakhstan Region, Akkayin District, Vlasovka	*Theileria orientalis*	28S rRNA	PZ312057
AKT-6399	Cattle	Aktobe Region, Oiyl District, Oiyl	*Theileria orientalis*	28S rRNA	PZ312058
EastK-9202	Cattle	East Kazakhstan Region, Ulan District, Aiyrtau	*Theileria orientalis*	28S rRNA	PZ312059
AKT-6392	Cattle	Aktobe Region, Oiyl District, Oiyl	*Theileria orientalis*	28S rRNA	PZ312060
ATR-6419	Cattle	Atyrau Region, Zhylyoi District, Kulsary	*Theileria orientalis*	28S rRNA	PZ312061
KZL-3214	Sheep	Kyzylorda Region, Kyzylorda District, Kyzylorda	*Theileria ovis*	28S rRNA	PZ312062
TRK-1271	Sheep	Turkistan Region, Baydibek District, Shayan	*Theileria annulata*	28S rRNA	PZ312064
ALM-7420	Sheep	Almaty Region, Karasay District, Karatobe	Theileria sp.OT3	28S rRNA	PZ312065
ALM-7420	Sheep	Almaty Region, Karasay District, Karatobe	*Theileria* sp.OT3	18S rRNA	PZ312066
