# Supplementary material for: Molecular epidemiology and geographic distribution of Theileria spp. in Kazakhstan: a countrywide cattle survey and a southern sheep survey
Source: Front Vet Sci. 2026 Oct 7;13:1987322. doi: 10.3389/fvets.2026.1987322 (PMC13683191; doi:10.3389/fvets.2026.1987322)
Supplement: Supplementary file 4 [file Data_Sheet_4.PDF]

Supplementary Table S4. Geographic associations between latitude and PCR detection of Theileria spp. in cattle based on generalized estimating equations.

Outcome	OR per 1° northward	95% CI	P
Any *Theileria* spp.	0.78	0.71–0.85	<0.001
T. annulata	0.70	0.65–0.77	<0.001
T. orientalis	0.90	0.82–0.99	0.038
T. sinensis	1.52	1.10–2.10	0.012
