# Supplementary material for: Molecular epidemiology and geographic distribution of Theileria spp. in Kazakhstan: a countrywide cattle survey and a southern sheep survey
Source: Front Vet Sci. 2026 Oct 7;13:1987322. doi: 10.3389/fvets.2026.1987322 (PMC13683191; doi:10.3389/fvets.2026.1987322)
Supplement: Supplementary file 5 [file Data_Sheet_5.PDF]

Supplementary Table S5. Reference Theileria and Babesia sequences used for phylogenetic analysis, including GenBank accession numbers, gene markers, hosts, and countries of origin.

No.	GenBank accession	Species	Gene	Host	Country
1	AF218825.1	*Theileria parva*	28S rRNA	*Syncerus caffer*	Kenya
2	BLIY01000007	*Babesia ovis*	28S rRNA	*Ovis aries*	Turkey
3	JN391432.1	*Babesia motasi*	28S rRNA	*Ovis aries*	China
4	JN696669.1	*Theileria luwenshuni*	28S rRNA	*Ovis aries*	China
5	JN696672.1	*Theileria ovis*	28S rRNA	*Ovis aries*	China
6	JN696673.1	*Theileria sinensis*	28S rRNA	*Bos taurus*	China
7	JN696674.1	*Theileria uilenbergi*	28S rRNA	*Ovis aries*	China
8	JN696681.1	*Theileria sinensis*	28S rRNA	*Bos taurus*	China
9	L28037.1	*Theileria taurotragi*	28S rRNA	*Damaliscus lunatus*	South Africa
10	U03602.2	*Theileria parva*	28S rRNA	*Syncerus caffer*	Kenya
11	NC_007344.1	*Theileria parva*	28S rRNA	*Bos taurus*	Kenya
12	NC_010575.1	*Babesia bovis*	28S rRNA	*Bos taurus*	USA
13	NC_011100.1	*Theileria annulata*	28S rRNA	*Bos taurus*	Turkey
14	NC_021366.1	*Theileria equi*	28S rRNA	*Equus caballus*	USA
15	NC_025260.1	*Theileria orientalis*	28S rRNA	*Bos taurus*	Japan
16	NC_027217	*Babesia bigemina*	28S rRNA	*Bos taurus*	Mexico
17	NW_021638340.1	*Babesia ovata*	28S rRNA	*Bos taurus*	Japan
18	NW_021639230.1	*Babesia* sp. Xinjiang	28S rRNA	*Ovis aries*	China
19	NW_027150539.1	*Babesia caballi*	28S rRNA	*Equus caballus*	USA
20	CCSG02000039.1	*Babesia divergens*	28S rRNA	Homo sapiens	France
21	CP141527	*Babesia gibsoni*	28S rRNA	*Canis lupus familiaris*	China
22	JBNLZY010000004.1	*Theileria haneyi*	28S rRNA	*Equus caballus*	USA
23	AB602880.1	*Theileria sp. Iwate 2-1*	18S rRNA	*Capricornis crispus*	Japan
24	AF097993.1	*Theileria velifera*	18S rRNA	*Bos taurus*	Tanzania
25	AF158710.1	*Theileria sp. BK115*	18S rRNA	*Gazella dama mhorr*	USA
26	AF162432.1	*Theileria sp.*	18S rRNA	*Odocoileus virginianus*	USA
27	AF162433.1	*Theileria sp. / T. cervi*	18S rRNA	*Odocoileus virginianus*	USA
28	AF245279.1	*Theileria youngi*	18S rRNA	*Neotoma fuscipes*	USA
29	AY260172.1	*Theileria ovis*	18S rRNA	*Ovis aries*	Turkey
30	AY260175.1	*Theileria separata*	18S rRNA	*Ovis aries*	South Africa
31	AY262115.1	*Theileria sp. China 1*	18S rRNA	*Bos taurus*	China
32	AY533143.1	*Theileria sp. OT1*	18S rRNA	*Ovis aries*	Spain
33	AY748462.1	*ia sp. Malelane sable an*	18S rRNA	*Hippotragus niger*	South Africa
34	AY748465.1	*Theileria sp. Mossel Bay 4*	18S rRNA	*Tragelaphus strepsiceros*	South Africa
35	AY748466.1	*Theileria sp. grey duiker 5*	18S rRNA	*Sylvicapra grimmia*	South Africa
36	BLIY01000007	*Babesia ovis*	18S rRNA	*Ovis aries*	Turkey
37	DQ104610.1	*Theileria sp. Hubei cattle*	18S rRNA	*Bos taurus*	China
38	DQ256380.1	*leria sp. ex Macheng buf*	18S rRNA	Buffalo	China
39	DQ286801.1	*ileria sp. ex Hongan buff*	18S rRNA	Buffalo	China
40	DQ286802.1	*ria sp. ex Macheng black*	18S rRNA	Black goat	China
41	DQ866839.1	*Theileria sp. OT3*	18S rRNA	*Rupicapra pyrenaica prenaica*	Spain
42	FJ213582.1	*Theileria sp. giraffe 0405*	18S rRNA	*Giraffa camelopardalis*	South Africa
43	FJ213586.1	*Theileria cf. mutans 2*	18S rRNA	*Syncerus caffer*	South Africa
44	FJ668368.1	*Theileria sp. HN1*	18S rRNA	*Hydropotes inermis argyropus*	South Korea
45	FJ668374.1	*Theileria sp. HN7*	18S rRNA	*Mazama gouazoubira*	Brazil
46	GQ292536.1	*Theileria sp. A2*	18S rRNA	Chinese water deer	China
47	HM538203.1	*Theileria sinensis*	18S rRNA	Buffalo	China
48	HQ184406.1	*Theileria cervi*	18S rRNA	Sika deer	China
49	HQ264133.1	*Theileria sp. H8*	18S rRNA	*Odocoileus virginianus*	USA
50	HQ844673.1	*Theileria sp. SZH-human*	18S rRNA	*Homo sapiens*	China
51	JF719835.1	*Theileria uilenbergi*	18S rRNA	*Ovis aries*	China
52	JN086222.1	*Theileria sp. 1 PJH-2012*	18S rRNA	*Rangifer tarandus tarandus*	USA
53	JN572700.1	*Theileria sp. B15a*	18S rRNA	*Syncerus caffer*	South Africa
54	JQ037786.1	*eileria cf. sinensis MC-20*	18S rRNA	*Syncerus caffer*	South Africa
55	JQ928907.1	*Theileria sp. NG-2012b*	18S rRNA	Giraffe	Kenya
56	JQ928924.1	*Theileria sp. NG-2012b*	18S rRNA	Giraffe	Kenya
57	JX134575.1	*Theileria capreoli*	18S rRNA	White-lipped deer	China
58	KC735165.1	*Theileria luwenshuni*	18S rRNA	*Ovis aries*	China
59	KF470868.1	*Theileria sp. OT3*	18S rRNA	*Ovis aries*	China
60	KF597064.1	*Theileria sp. NG-2013c*	18S rRNA	Waterbuck	Kenya
61	KF597090.1	*Theileria sp. NG-2013b*	18S rRNA	Giraffe	Kenya
62	KU647704.1	*Theileria haneyi*	18S rRNA	*Equus caballus*	USA
63	KY953226.1	*Theileria ornithorhynchi*	18S rRNA	*Ornithorhynchus anatinus*	Australia
64	L19082.1	*Theileria taurotragi*	18S rRNA	*Taurotragus oryx*	South Africa
65	LC863650.1	*Theileria bicornis*	18S rRNA	*Panthera pardus*	Nepal
66	MN493109.1	*Theileria sp. OT3*	18S rRNA	*Ovis aries*	Turkey
67	OR067885.1	*Theileria sp.*	18S rRNA	Buffalo	India
68	OR067887.1	*Theileria sp.*	18S rRNA	Buffalo	India
69	OR067888.1	*Theileria sp.*	18S rRNA	Buffalo	India
70	OR067889.1	*Theileria sp.*	18S rRNA	Cattle	India
71	PV174930.1	*Theileria sp.*	18S rRNA	Goat	India

No.	GenBank accession	Species	Gene	Host	Country
72	CCSG02000039	*Babesia divergens*	18S rRNA	*Homo sapiens*	France
73	CP141526	*Babesia gibsoni*	18S rRNA	*Canis lupus familiaris*	China
74	NC_007344.1	*Theileria parva*	18S rRNA	*Bos taurus*	Kenya
75	NC_010575.1	*Babesia bovis*	18S rRNA	*Bos taurus*	USA
76	NC_011100.1	*Theileria annulata*	18S rRNA	*Bos taurus*	Turkey
77	NC_021366.1	*Theileria equi*	18S rRNA	*Equus caballus*	USA
78	NC_025260.1	*Theileria orientalis*	18S rRNA	*Bos taurus*	Japan
79	NC_027217	*Babesia bigemina*	18S rRNA	*Bos taurus*	Mexico
80	NW_021638340.1	*Babesia ovata*	18S rRNA	*Bos taurus*	Japan
81	NW_021639230.1	*Babesia* sp. Xinjiang	18S rRNA	*Ovis aries*	China
82	NW_027150541	*Babesia caballi*	18S rRNA	*Equus caballus*	USA
83	D87191.1	*Theileria sp. CB10*	MPSP	*Bos taurus*	South Korea
84	AB218442.1	*heileria sp. Okinawa C5-*	MPSP	*Bos taurus*	Japan
85	AB469171.1	*Theileria orientalis*	MPSP	*Bos taurus*	Japan
86	AB469173.1	*Theileria orientalis*	MPSP	*Bos taurus*	Japan
87	AB469175.1	*Theileria orientalis*	MPSP	*Bos taurus*	Japan
88	AB560830.1	*Theileria orientalis*	MPSP	*Bubalus arnee*	Viet Nam
89	AB560833.1	*Theileria orientalis*	MPSP	*Ovis aries*	Viet Nam
90	AB562561.1	*Theileria orientalis*	MPSP	*Bubalus arnee*	Thailand
91	AB690866.1	*Theileria orientalis*	MPSP	*Bos taurus*	Sri Lanka
92	AB690870.1	*Theileria orientalis*	MPSP	*Bos taurus*	Sri Lanka
93	AB871369.1	*Theileria orientalis*	MPSP	*Bos sp.*	Myanmar
94	AB917303.1	*Theileria orientalis*	MPSP	*Bos taurus*	Egypt
95	AB917305.1	*Theileria orientalis*	MPSP	*Bos taurus*	Egypt
96	AB917306.1	*Theileria orientalis*	MPSP	*Bubalus bubalis*	Egypt
97	AF236095.1	*Theileria buffeli*	MPSP	*Bos taurus*	Australia
98	D87202.1	*Theileria sp. DJ2*	MPSP	*Bos taurus*	South Korea
99	D87205.1	*Theileria sp. OK3*	MPSP	*Bos taurus*	Japan
100	FJ560982.1	*Theileria sp. JN51*	MPSP	cattle	South Korea
101	FJ560983.1	*Theileria sp. HpTick*	MPSP	tick	South Korea
102	FJ560985.1	*Theileria sp. CJ5R-26*	MPSP	cattle	South Korea
103	FJ560987.1	*Theileria sp. YB7*	MPSP	cattle	South Korea
104	GQ180189.1	*Theileria sinensis*	MPSP	yak	China
105	GQ180193.1	*Theileria sinensis*	MPSP	yak	China
106	GQ180196.1	*Theileria sinensis*	MPSP	cow	China
107	JN252703.1	*Theileria orientalis*	MPSP	*Bos taurus*	Australia
108	JN648699.1	*Theileria sergenti*	MPSP	grazing cattle	South Korea
109	KM043772.1	*Theileria orientalis*	MPSP	*Bubalus bubalis*	India
110	KM624619.1	*Theileria orientalis*	MPSP	*Bos taurus*	Australia
111	KM624620.1	*Theileria orientalis*	MPSP	*Bos taurus*	Australia
112	KM624621.1	*Theileria orientalis*	MPSP	*Bos taurus*	Australia
113	KP347560.1	*Theileria orientalis*	MPSP	cattle	Kenya
114	KP347561.1	*Theileria orientalis*	MPSP	cattle	Kenya
115	KT220519.1	*Theileria orientalis*	MPSP	*Bos taurus*	Turkey
116	KU170784.1	*Theileria orientalis*	MPSP	cattle	Ethiopia
117	KU170786.1	*Theileria orientalis*	MPSP	cattle	Ethiopia
118	KU764501.1	*Theileria orientalis*	MPSP	cattle	Thailand
119	KU764503.1	*Theileria orientalis*	MPSP	cattle	Thailand
120	KU764504.1	*Theileria orientalis*	MPSP	cattle	Thailand
121	KY495506.1	*Theileria orientalis*	MPSP	*Bos taurus*	China
122	KY495508.1	*Theileria orientalis*	MPSP	*Bos taurus*	China
123	LC125423.1	*Theileria orientalis*	MPSP	*Bos taurus*	Viet Nam
124	LC125428.1	*Theileria orientalis*	MPSP	*Bos taurus*	Viet Nam
125	LC125430.1	*Theileria orientalis*	MPSP	*Bos taurus*	Viet Nam
126	LC125431.1	*Theileria orientalis*	MPSP	*Bos taurus*	Viet Nam
127	LC125432.1	*Theileria orientalis*	MPSP	*Bubalus bubalis*	Viet Nam
128	LC125439.1	*Theileria orientalis*	MPSP	*Bubalus bubalis*	Viet Nam
129	LC125441.1	*Theileria orientalis*	MPSP	*Bubalus bubalis*	Viet Nam
130	LC341308.1	*Theileria sp. NY-2017*	MPSP	*Cervus nippon yesoensis*	Japan
131	LC341346.1	*Theileria sp. NY-2017*	MPSP	*Cervus nippon yesoensis*	Japan
132	LC341392.1	*Theileria sp. NY-2017*	MPSP	*Ixodes persulcatus*	Japan
133	LC341393.1	*Theileria sp. NY-2017*	MPSP	*Ixodes persulcatus*	Japan
134	LC341394.1	*Theileria sp. NY-2017*	MPSP	*Ixodes persulcatus*	Japan
135	LC341395.1	*Theileria sp. NY-2017*	MPSP	*Haemaphysalis japonica*	Japan
136	LC438466.1	*Theileria orientalis*	MPSP	*Bos taurus*	Sri Lanka
137	LC438471.1	*Theileria orientalis*	MPSP	*Bos taurus*	Sri Lanka
138	LC438486.1	*Theileria orientalis*	MPSP	*Bos taurus*	Sri Lanka
139	LC661355.1	*Theileria orientalis*	MPSP	*Bos taurus*	Egypt
140	LC661356.1	*Theileria orientalis*	MPSP	*Bos taurus*	Egypt
141	MF145155.1	*Theileria orientalis*	MPSP	dairy cattle	China
142	MF145156.1	*Theileria orientalis*	MPSP	dairy cattle	China
143	MF145157.1	*Theileria orientalis*	MPSP	dairy cattle	China
144	MF145158.1	*Theileria sergenti*	MPSP	dairy cattle	China

No.	GenBank accession	Species	Gene	Host	Country
145	MH539825.1	*Theileria orientalis*	MPSP	cattle	China
146	MH539826.1	*Theileria orientalis*	MPSP	cattle	China
147	MH539827.1	*Theileria orientalis*	MPSP	cattle	China
148	MH539828.1	*Theileria orientalis*	MPSP	cattle	China
149	MH539829.1	*Theileria orientalis*	MPSP	cattle	China
150	MH539831.1	*Theileria orientalis*	MPSP	cattle	China
151	MH539832.1	*Theileria orientalis*	MPSP	cattle	China
152	MK436215.1	*Theileria orientalis*	MPSP	*Rhipicephalus microplus*	China
153	MK436238.1	*Theileria orientalis*	MPSP	*Rhipicephalus microplus*	China
154	MN630024.1	*Theileria sinensis*	MPSP	cattle	China
155	MT240816.1	*Theileria sinensis*	MPSP	cattle	Malaysia
156	MT240817.1	*Theileria sinensis*	MPSP	cattle	Malaysia
157	MT891280.1	*Theileria orientalis*	MPSP	cattle	South Korea
158	MW752894.1	*Theileria orientalis*	MPSP	dairy cow	China
159	MZ068224.1	*Theileria orientalis*	MPSP	buffalo	India
160	MZ068225.1	*Theileria orientalis*	MPSP	buffalo	India
161	MZ852008.1	*Theileria orientalis*	MPSP	cattle	Russia
162	OM830307.1	*Theileria orientalis*	MPSP	goat	Thailand
163	ON490799.1	*Theileria orientalis*	MPSP	cattle	Kazakhstan
164	ON490800.1	*Theileria orientalis*	MPSP	cattle	Kazakhstan
165	ON490812.1	*Theileria orientalis*	MPSP	cattle	Kazakhstan
